# Polyphenols and Phenolic Glucosides in Antibacterial Twig Extracts of Naturally Occurring *Salix myrsinifolia* (Salisb.), *S. phylicifolia* (L.) and *S. starkeana* (Willd.) and the Cultivated Hybrid *S. x pendulina* (Wender.)

**DOI:** 10.3390/pharmaceutics16070916

**Published:** 2024-07-09

**Authors:** Enass Salih, Eunice Ego Mgbeahuruike, Stella Prévost-Monteiro, Nina Sipari, Henry Väre, Brigita Novak, Riitta Julkunen-Tiitto, Pia Fyhrqvist

**Affiliations:** 1 Division of Pharmaceutical Biosciences, Faculty of Pharmacy, University of Helsinki, 00100 Helsinki, Finland; eunice.mgbeahuruike@helsinki.fi (E.E.M.); pia.fyhrquist@helsinki.fi (P.F.); 2 Faculty of Pharmaceutical Sciences, University of Bordeaux, 33076 Bordeaux, France; stella.prevost-monteiro@etu.u-bordeaux.fr; 3 Viikki Metabolomics Unit, Organismal and Evolutionary Biology Research Programme, Faculty of Biological and Environmental Sciences, University of Helsinki, 00100 Helsinki, Finland; nina.sipari@helsinki.fi; 4 Botanical Museum, Finnish Museum of Natural History, University of Helsinki, 00100 Helsinki, Finland; henry.vare@helsinki.fi; 5 Faculty of Pharmacy and Biochemistry, University of Zagreb, 10000 Zagreb, Croatia; bnovak@pharma.hr; 6 Department of Environmental and Biological Sciences, Faculty of Science and Forestry, University of Eastern Finland, 80100 Joensuu, Finland; riitta.julkunen-tiitto@uef.fi

**Keywords:** *Salix* spp. twigs, proanthocyanidins, procyanidins, salicinoids, flavonoids, phenolic glucosides antibacterial effect, UPLC/QTOF-MS

## Abstract

(1) Background: *Salix* species occurring in Finland have not been well studied for their antimicrobial potential, despite their frequent use for lung and stomach problems in traditional medicine. Thus, twig extracts of three species of *Salix* that are found naturally in Finland and one cultivated species were screened for their antimicrobial properties against human pathogenic bacteria. *S. starkeana* and *S. x pendulina* were screened for antibacterial effects for the first time. (2) Methods: An agar diffusion and a microplate method were used for the screenings. Time-kill effects were measured using a plate-count and a microplate method. A DPPH-method using a qualitative TLC-analysis was used to detect antioxidant compounds in antimicrobial extracts. Metabolites from a *S. myrsinifolia* extract showing good antibacterial effects were identified using UPLC/QTOF-MS. (3) Results: A methanol extract of *S. starkeana* was particularly active against *B. cereus* (MIC 625 µg/mL), and a methanol extract of *S. myrsinifolia* showed good activity against *S. aureus* and *B. cereus* (MIC 1250 µg/mL) and showed bactericidal effects during a 24 h incubation of *B. cereus*. Moreover, a decoction of *S. myrsinifolia* resulted in good growth inhibition against *P. aeruginosa*. Our UPLC/QTOF-MS results indicated that proanthocyanidins (PAs), and especially the dimer procyanidin B1 (*m*/*z* 577) and other procyanidin derivatives, including highly polymerized proanthocyanidins, were abundant in *S. myrsinifolia* methanol extracts. Procyanidin B1 and its monomer catechin, as well as taxifolin and p-hydroxycinnamic acid, all present in *S. myrsinifolia* twigs, effectively inhibited *B. cereus* (MIC 250 µg/mL). (4) Conclusions: This study indicates that Finnish *Salix* species contain an abundance of antibacterial condensed tannins, phenolic acids and other polyphenols that deserve further research for the antibacterial mechanisms of action.

## 1. Introduction

Bacterial infections are a significant cause of mortality and morbidity globally due to the growing antimicrobial resistance, including multi-drug-resistant bacterial strains [1]. The WHO considers antimicrobial resistance to be one of the most urgent issues for medical science [2]. However, since the 1990s, the number of new antibiotic agents has been on a sharp decline, and most new antibiotics are developed from molecular structures of existing antibiotics [3,4]. Plants produce a vast chemical diversity of secondary compounds often in response to biotic stress, such as herbivory, weeds, pathogenic fungi and bacteria, and abiotic stress, such as temperature and ultraviolet radiation [5]. Thus, plants could be good sources for novel antimicrobial extracts and compounds. Plant-derived compounds might have different mechanisms of action when compared to conventional antibiotics, and in addition, plant extracts and compounds have resistance-modifying activity in combinations with antibiotics [6,7]. Thus, plant-derived compounds and extracts, either alone or in combination with antibiotics, could have significance for the treatment of multi-drug resistant bacteria [8]. The World Health Organization (WHO) has described medicinal plants as one of the potential sources of new anti-infectious agents [9,10], opening the door for interdisciplinary innovative research based on traditional knowledge. Moreover, medicinal plants remain the most abundant natural primary source of antimicrobial agents with more than 1340 plants having reported antimicrobial activity, including some *Salix* spp., and more than 30,000 isolated and structurally elucidated molecules, of which many have been shown to have good antibacterial potential [11].

*Salix* species or willows belong to the family Salicaceae. Willows are also known as sallows and osiers, and about 500 species are known, mainly from the northern hemisphere [12]. In Finland, 24 *Salix* species are native [13]. Willows are deciduous and dioecious (male and female inflorescences on separate plants) trees, shrubs or dwarf shrubs growing predominantly in the temperate and arctic regions of the world in a wide range of moist or wet habitats, such as moist forests, forest bogs, shrub thickets, meadows, lake shores, riversides, mountains (alpine and subnival) and roadsides [14,15,16].

In Finland and Sweden, willows, including *S. viminalis*, *S. x dasyclados*, *S.* cv. *Aquatica* and *S. myrsinifolia*, are commonly cultivated for energy, extractives and colorant production as short rotation woody crops (SRWCs) on mined peatlands [17,18]. In addition, willows are used as wind shelters to prevent erosion in a sustainable agricultural forestry “agroforestry system” as forest farming [16,19].

*Salix* bark is well-known for its ethno-medicinal uses for the treatment of fever and aches, with a history back to the Greek, Assyrian, and Egyptian civilizations [20]. In addition, *Salix* spp. bark and leaf decoctions were used in traditional medicine for the treatment of lung problems, bedsores and gastrointestinal problems, which indicates that willows contain antimicrobial compounds [21]. Moreover, in many Scandinavian countries, various parts of the *Salix* spp. have been used as food additives and supplements, either raw or cooked, although willows have a bitter taste, even though some species are sweeter and taste like watermelon or cucumber [22,23]. In the European Union Herbal Monograph [24] and according to the European Union Pharmacopeia [25], the bark of three willow species, *S. alba*, *S. daphnoides* and *S. purpurea*, is described to be used as a quantified dry herbal preparation (tablets) of a 70% aqueous EtOH or an aqueous extract for the treatment of fever associated with common cold and other disorders, as well as for mild headache and lower back pain. In this herbal preparation, salicin and other salicylates that metabolize to salicylic acid are thought to be one of the main active ingredients, in addition to other polyphenols, such as condensed tannins (the proanthocyanidins) and flavonoids [26]. Moreover, another component, triandrin found in the bark of certain *Salix* species, has been considered for the treatment of rheumatic conditions [27].

Previous research on *Salix* spp. focused especially on their phenolic glucosides, flavonoids and proanthocyanidins (condensed tannins) in relation to abiotic and biotic environmental factors, chemotaxonomy, phenology and anti-inflammatory effects [14,26,28,29,30,31,32]. A few studies were also performed on lignans and triterpenes in *Salix* spp. [33,34]. Recently, research on *Salix* phytochemistry and bioactivity, including the antimicrobial potential of willow extracts and compounds, has gained new interest. For example, the *Salix* hybrid “Klara” (*Salix burjatica* × *S. viminalis*) × *S. burjatica* and yarns made from this hybrid were found to possess promising growth-inhibitory and anti-biofilm effects against *S. aureus* [35,36]. Altogether, both polar and lipophilic extracts of various *Salix* species were found to possess antibacterial, antifungal, anti-virulence and anti-quorum sensing properties [37,38,39,40]. However, studies on the antimicrobial properties of some willow species that are commonly growing in Finland and other Nordic countries, such as *S. phylicifolia* and *S. myrsinifolia*, are still scarce. Significantly, there are no previous studies on the antibacterial effects of *S. starkeana* and *S. x pendulina* that we have been included in this present research. In addition, clones of *S. phylicifolia* and *S. myrsinifolia* that were tested for suitability in short-rotation cultivation in Finland were found to be resistant to pests, including the rust fungus *Melampsora* spp. [18]. This and the traditional medicinal use of *Salix* for symptoms related to bacterial infections and for the treatment of infected wounds [21] would indicate that willows contain valuable antimicrobial compounds. Due to this, in our present research, we evaluated the antibacterial effects of extracts made from dormant twig material of the naturally occurring *S. myrsinifolia*, *S. starkeana* and *S. phylicifolia* and the cultivated hybrid *S. x pendulina*. In addition, UPLC/QTOF-MS was used for deciphering the molecular masses of chemical constituents in a methanol extract of *S. myrsinifolia* that showed good antimicrobial activity in our screenings.

## 2. Materials and Methods

### 2.1. Plant Collections

**Figure 1:**
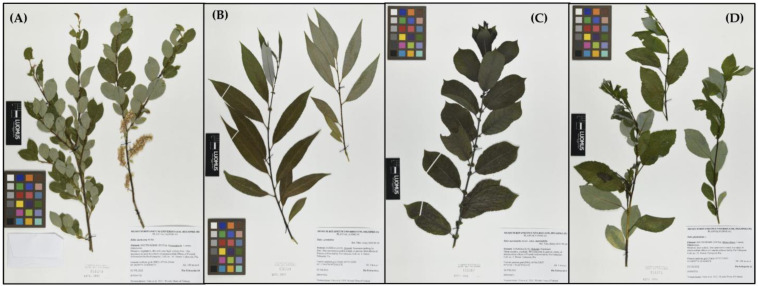
(**A**) *Salix starkeana* No. H856578 with female catkins, (**B**) *S. x pendulina* No. H856569, (**C**) *S. myrsinifolia* No. H856575 and (**D**) *S. phylicifolia* No. H856576 are deposited as voucher specimens at the Botanical Museum, Finnish Museum of Natural History, University of Helsinki, Finland. Photo: Jaana Haapala.

Dormant young twigs from all naturally occurring willows used in our study, e.g., *Salix starkeana*, *S. myrsinifolia* and *S. phylicifolia*, were collected from the Lammi region, Finland in April 2022. The cultivated hybrid *S. x pendulina* is grown as an ornamental tree in Helsinki, and the dormant young twigs were collected in February 2022 from a stand of trees growing on Seurasaarentie. Moreover, after the leaf burst Voucher samples were collected from each specimen (Figure 1), all *Salix* samples, including the hybrid, were identified by the Senior Curator, Dr. Henry Väre, Botanical Museum, Finnish Museum of Natural History, University of Helsinki, and voucher specimens were given herbarium numbers. The voucher numbers and collection site coordinates (in brackets) of the studied *Salix* species in this paper are as follows: *S. myrsinifolia* No. H856575 (61.05195° N 25.03185° E); *S. starkeana* No. H856578 (61.06234° N 25.04263° E); *S. x pendulina* No. H856569 (60.11186° N 24.53105° E) and *S. phylicifolia* No. H856576 (61.05260° N 25.03341° E). All voucher samples have been deposited in the Botanical Museum, Finnish Museum of Natural History, University of Helsinki, Finland (Figure 1).

The dormant *Salix* twigs were dried at room temperature for three to four weeks and then grinded using a dry grinder machine (AURUM-PHARMAKON Oy Apta Ab, PELITEOS Oy No 7018, Helsinki, Finland). The resulting plant powders were stored in paper bags.

### 2.2. Extraction Methods

#### 2.2.1. Water Extracts

According to the method described in Salih et al. [41], Milli-Q water was used to prepare decoctions and macerations of the powdered twigs of the *Salix* species. For the decoctions, five grams of the plant powder was mixed with 150–200 mL distilled water and was brought to a boil for five minutes, whereafter the extraction was continued overnight. The weight-to-volume ratio (plant material:solvent) for the macerations was the same as for the decoctions, but extraction was performed at room temperature during the whole extraction time. During the overnight extraction, a magnetic stirrer was used to facilitate the extraction (RCT, digital, Staufen, Germany). After completing the extraction, the extracts were centrifuged at 3000 rpm for 15 min in Eppendorf AG centrifuge tubes 5810 R (volume 50 mL, Hamburg, Germany). Both maceration and decoction extracts were stored at −20 °C, whereafter they were dried using a freeze dryer for three days (SCANVAC Coolsafe 110-4 Pro, Labogene, Denmark; Vacuum Pump RZ 2.5, GMBH, Hamburg, Germany). Both extracts were dissolved in methanol at 50 mg/mL for antibacterial tests.

#### 2.2.2. Methanol Extracts

Twenty grams of powdered twig material from the studied *Salix* species was extracted using a volume of 400–500 mL of 100% methanol. The extractions were performed overnight, using a magnetic stirrer (RCT, digital, Staufen, Germany). The methanol was evaporated using a rotary evaporator (Heidolph VV2000, Schwabach, Germany) and a freeze drier (SCANVAC Coolsafe 110-4Pro, Lillerød, Denmark; Vacuum Pump RZ 2.5, GMBH, Hamburg, Germany) for three days. The dried extracts were dissolved in methanol at 50 mg/mL stock solutions for the antibacterial screenings.

Extraction yields were calculated for all extracts as follows:

% Extraction yield = dry weight of extract/dry weight of the plant material × 100 (1)

### 2.3. Antimicrobial Assays

#### 2.3.1. Bacterial Strains and Pure Compounds

*Bacillus cereus* ATCC 10987, *Staphylococcus aureus* ATCC 25923, *Pseudomonas aeruginosa* ATCC 27853 and *Escherichia coli* 673/3 ATCC P25922 were used as model ATCC bacterial strains.

#### 2.3.2. Agar Diffusion Assays

Dormant twigs of *Salix x pendulina*, *S. myrsinifolia*, *S. starkeana* and *S. phylicifolia* were screened for their antibacterial effects using an agar diffusion method as described in Fyhrquist et al. [42] and Salih et al. [43]. A few colonies from the slants containing the tested bacteria were transferred to 25 mL of Mueller-Hinton broth and incubated overnight in an orbital shaker (Stuart, SI500, Holsworthy, UK) at +37 °C, 200 RPM. According to the measured optical density (OD) at the wavelength at 625 nm, using a Multiskan Sky spectrophotometer (Thermo Fisher Scientific, Vantaa, Finland), the bacterial suspensions were diluted using Mueller-Hinton broth to achieve an absorbance (A_620_) of 0.1 (≈1 × 10^8^ CFU/mL). Two hundred microliters of the inoculum was pipetted onto the Petri dishes (∅ = 14 cm) containing 25 mL base agar as a bottom layer and 25 mL iso-sensitest agar as a top layer, whereafter wells were made in the agar using a cork borer. Two hundred microliters of plant extracts (50 mg/mL), antibiotics and pure compounds (10 mg/mL) was pipetted into the well. The Petri dishes were incubated overnight at +37 °C (memmert oven, GmbH, Hamburg, Germany). Experiments were performed in triplicate, and the antibacterial effect was measured as the average of the triplicate inhibition zone diameters (IZDs) ± standard errors of means (SEMs).

Commercially available standard pure compounds that are present in *Salix* spp., such as *p*-hydroxy cinnamic acid (Sigma-Aldrich, Darmstadt, Germany), *m*-hydroxy cinnamic acid (Sigma-Aldrich), salicortin (Phytolab, Munich, Germany), Tremulacin (Sigma-Aldrich), procyanidin B1 (22411, Cayman, Ann Arbor, MI, USA), taxifolin (Cayman), catechin (Biopurify Chemicals Ltd., Chengdu, China) and naringenin (Biopurify Chemicals Ltd., Chengdu, China), were used for the antibacterial screening. Salireposide, salicyloyl-salicin, acetylsalicortin and salicin were isolated from *Salix* spp., and populin was isolated from *Populus tremula* (Salicaceae), and these were a kind gift from Emeritus Professor Riitta Julkunen-Tiitto, University of Eastern Finland, Joensuu.

#### 2.3.3. Turbidimetric Microplate Assay

The antibacterial activity as a percentage of growth inhibition and as MICs of the plant extracts was determined using sterile 96-well plates (Thermo Fisher Scientific) according to the method of Fyhrquist et al. [44] and Salih et al. [45]. Each sample and its control (well containing only plant extract and broth) were tested in duplicate. Any absorption at 620 nm resulting from the plant extract, antibiotic or pure compound itself was subtracted from the absorption of the corresponding plant extract, antibiotic or pure compound in bacterial suspension. Thus, in each test, altogether, four wells were filled with the same sample (extract, pure compound or antibiotic): two wells were filled with the sample and bacteria and two wells with the corresponding sample in broth. The four wells of each replicate were filled with 100 µL of plant extracts (from 39 to 2500 µg/mL), antibiotics or pure compounds (from 0.030 to 500 μg/mL) dissolved in Mueller-Hinton broth (MHB). In addition, 100 µL of the bacterial suspension containing 1 × 10^6^ CFU/mL was added to two duplicate wells (that were called test wells, GT), so that the final bacterial number in the wells was 5 × 10^5^ CFU/mL [46]. Moreover, the corresponding control wells, containing the same extract, antibiotic or pure compounds, were filled with 100 µL of Mueller-Hinton broth to serve as sample controls (SCs). Any absorption resulting from the sample control wells was subtracted from the corresponding test wells. Six wells in the second column of the microplate served as the growth control (GC) and were filled with 100 µL of the bacterial suspension at 1 × 10^6^ CFU/mL and 100 µL of the sterile Mueller Hinton-broth (MHB). Tetracycline, rifampicin and ciprofloxacin hydrochloride (Sigma-Aldrich) were used as positive controls. The microplates were incubated in a BioSan incubator (Thermo-Shaker PST-60HL-4) at 350 RPM, +37 °C, for 24 h. Subsequently, the optical density (OD620) was measured using a Multiskan Sky Microplate Spectrophotometer (Thermo Fisher Scientific). The minimum inhibitory concentration was considered as the lowest concentration of the extract, compounds or antibiotics that resulted in a growth inhibition of 90% or more (no visible growth) compared to the growth control (defined as 100% growth). The percentage growth inhibition was calculated as the mean percentage of growth of duplicates in relation to the growth of the growth control (GC) as described in the below equations:

(2) $$\mathrm{Percentage}\;(\%)\;\mathrm{bacterial}\;\mathrm{growth}=[((\bar{x}{\mathrm{GT}}_{A620}\;-\;\bar{x}{\mathrm{SC}}_{A620})/\bar{x}{\mathrm{GC}}_{A620})\;\times \;100]$$

(3) $$\mathrm{Percentage}\;(\%)\;\mathrm{inhibition}\;\mathrm{of}\;\mathrm{growth}=100\;(\%\;\mathrm{growth}\;\mathrm{of}\;\mathrm{the}\;\mathrm{growth}\;\mathrm{control})-[((\bar{x}{\mathrm{GT}}_{A620}\;-\;\bar{x}{\mathrm{SC}}_{A620})/\bar{x}{\mathrm{GC}}_{A620})\;\times \;100]$$

where $\bar{x}$ = the average of the duplicates of the studied wells; GT_A620_ = optical density of the test well (plant extracts, compounds or antibiotics incubated with the bacterium); SC_A620_ = optical density of the sample control wells (controls containing the plant extracts, compounds or antibiotics with broth only and no bacteria); GC_A620_ = optical density of the growth control.

#### 2.3.4. Statistical Analysis and Data Management

The data from agar diffusion, obtained as the diameters of inhibition zones, IZDs, were expressed as the mean of triplicates (*n* = 3) ± SEM, and the data were obtained from two individual experiments, while the data of the percentage inhibition of growth was expressed as the mean percentage of growth of duplicates (*n* = 2) ± SEM, obtained from three independent experiments.

#### 2.3.5. Microplate Method Measuring Optical Density Combined with a Plate Count Method to Assess Time-Kill Effects

A microplate method combined with plate counts, modified from Mordmuang et al. [47] and Baysal [48], was used to measure the growth-inhibitory effects of a *Salix myrsinifolia* twig methanol extract on the growth of *B. cereus* as a function of time. The protocol for the microplate assay was the same that we have used for percentage growth and MIC estimates, with an inoculum containing 1 × 10^6^ CFU/mL. The effects of the extract on bacterial growth were compared to a growth control and to the positive control, tetracycline. For the time-kill test, the concentrations of the plant extract and the antibiotic were chosen based on their MICs (4 × MIC–0.5 × MIC), where were obtained in our previous tests. Thus, for the *S. myrsinifolia* methanol extract with an MIC of 2500 µg/mL against *B. cereus*, concentrations of 5000, 2500, 1250 and 625 µg/mL were used. For tetracycline, giving an MIC of 0.48 µg/mL against *B. cereus*, the concentrations 0.48 and 0.24 µg/mL were used. The microplate was incubated at 37 °C, 350 RPM. Optical densities (OD620) were measured every other hour of the time-kill test beginning from time-point zero of the assay and altogether at five different time points (2, 4, 6, 8 and 24 h) using a Multiskan Sky Microplate Spectrophotometer (Thermo Fisher Scientific). A plate-count assay was performed in parallel with the optical density assay as follows: every second hour (at 0, 2, 4, 6, 8 and 24 h incubation), 10 µL was pipetted from the microplate wells and diluted with broth for plate counts (1:10×, 100×, 1000× and 10,000× dilutions were made). For the growth control, dilutions starting from 100×, 1000×, 10,000× and 100,000× werse made. Then, 50 µL of each dilution was plated on two replicate Petri dishes (9 mm) that contained Mueller-Hinton agar. This dilution and plating procedure were repeated at each sampling point. After incubation for 24 h at 37 °C, the countable number of bacterial colonies (between 30 and 300 CFU/mL) was counted using a manual colony counter available in a Petri dish magnifier (C-110, New Jersey, NJ, USA). All time-kill experiments were performed in duplicate.

The colony forming units (CFU/mL) were calculated according to the below equation:

CFU/mL = (average number of colonies formed on duplicate plates × dilution factor) volume of culture plated in mL(4)

### 2.4. Phytochemical Assays

#### UPLC/QTOF-MS Analysis

A methanol extract of *S. myrsinifolia* dormant twigs was dissolved in 1 mL of 50% methanol (HPLC grade) to a concentration of 5 mg/mL for UPLC/QTOF-MS analysis. A standard mixture containing salicin, salicortin (86064, Phytolab, Munich, Germany), luteolin (Sigma-Aldrich), naringenin (Biopurify Chemicals Ltd., Chengdu, China), taxifolin (18647, Cayman), acetylsalicortin, procyanidin-B (22411, Cayman) and catechin (Biopurify Chemicals Ltd., Chengdu, China) was used as a standard. Acquity Premier UPLC liquid chromatography (Waters Corporation, Milford, MA, USA), connected to the HDMS mass spectrometer (Synapt G2 HDMS; Waters Corporation, Milford, MA, USA) via an Acquity PDA detector (Waters Corporation, Milford, MA, USA), was used. The electrospray ionization (ESI) in resolution mode was used in both negative and positive modes according to a method described in Sipari et al. [49] with a slight modification. The mass ranges of 100–1000 *m*/*z* (ESI^−^) and *m*/*z* 100–1600 in (ESI^+^) were employed. The scanning time was set to 0.1 s. A reverse-phase column, Waters Acquit BEH C18 (50 × 2.1 mm, Ø 1.7 µm), at a temperature of +40 °C was used for the runs. Solvent system A was H_2_O with 0.1% formic acid (Sigma-Aldrich), and solvent system B was ACN (Sigma-Aldrich) with 0.1% formic acid (Sigma-Aldrich). Linear gradient elution was performed from 5% B to 95% B in 9 min, switched back to 5% B and left to stabilize for 1 min; thus, the total run time was 10 min. A photodiode array detector (PDA detector, Darmstadat, Germany) was adjusted at 40 points/sec, and the UV wavelength was at 210–500 nm. The compounds in the extracts were identified based on their mass spectra (*m*/*z*) and fragmentation patterns partly by using an available compound library, internal standards and MassLynx 4.1 software (Waters Corporation, Milford, MA, USA) and partly by comparing the spectra obtained to mass spectra of the injected standard compounds salicortin, luteolin, naringenin, taxifolin, procyanidin-B and catechin.

The mass error was estimated as a measure of the accuracy of the mass measurement, so that the measured molecular weight of a compound (M_measured_) was compared to the calculated molecular weight (M_calculated_) of this same compound. The mass error is described as parts per million (ppm) and is calculated according to the below formula:

PPM (ppm, parts per million mass error) mass accuracy = (M_measured_ − M_calculated_) × 10^6^/M_calculated_ (5)

where M_measured_ = measured mass in QTOF-MS; M_calculated_ = exact calculated mass according to the CAS molecular formula of the identified compound. The molecular weight of the hydrogen atom (1.0078) was subtracted from all the calculated masses to achieve the negative ions [M–H]. Exact molecular weights were calculated according to monoisotopic masses for the carbon atom of 12.000, the oxygen atom of 15.9949 and the hydrogen atom of 1.0078.

### 2.5. Qualitative Antioxidant Analysis of S. starkeana Extracts Using a TLC and DPPH Assay

An evaluation of the antioxidant capacity of the maceration and methanol extracts of *Salix starkeana* was performed according to Salih et al. [45]. Reversed-phase aluminum-backed silica plates RP-18F254 (Merck, Darmstadt, Germany) and the DPPH reagent were used to detect the antioxidant compounds and fractions in the *Salix starkeana* extracts.

A total of 20 µL of extract (50 mg/mL) and 10 µL of compounds (5 mg/mL) was applied equidistantly in the TLC plate (10 × 10 cm, RP-18 F254s Merck, Darmstadt, Germany). Triandrin, salicortin and salicin were used as reference compounds, since they are known to be present in many *Salix* spp. Methanol, water and orthophosphoric acid (50:50:1; *v*:*v*:*v*) were used as the eluent. The fractionated spots were visualized using a Camaq Reprostar UV-detector at two different fluorescence and quenching UV wavelengths (366 and 254 nm respectively), and a smartphone was used to photograph the eluted TLC. The developed TLC plates containing the maceration and methanol extract of the *S. starkeana* twigs were subjected to qualitative antioxidant analysis using 0.2% *w*/*v* of the DPPH reagent in methanol (2,2-Diphenyl-1-picrylhydrazyl, D9132-1G, SigmaAldrich Schnelldorf, Germany). All compounds with antioxidant properties were visible as yellow spots in daylight due to the reaction of the DPPH radicals (violet) with the antioxidant compounds.

## 3. Results

### 3.1. Description of the Collected Species

In our present research, the dormant twig extracts were chosen for the antibacterial screening, since willow dormant twigs and buds are known to contain an abundance of secondary compounds, including condensed tannins, that could play an important part of the antibacterial activity of the *Salix* plants [50,51]. Our collected willow species are presented below. Moreover, since *Salix* species identification is challenging from dormant twigs, additional field work was performed after the flower and leaf burst, in May and June 2022, to identify the *Salix* species correctly using the catkin (flower) and leaf morphology. Thus, we also present these species characteristics beneath.

#### 3.1.1. Pale Willow-*Salix starkeana* subs. *starkeana* (Willd.), Voucher No. 856578

Our specimen of pale willow (Figure 1A) was a female shrub (2.5 m) growing on a dry meadow in Lammi adjacent to a line of high-voltage power lines, which is a typical growth place in Finland for this willow species. During our winter collection in April 2022, this species was distinguished from the rest of the vegetation (mostly *Betula pubescens*) by its slender, bright red new annual shoots with reddish-brown buds. In Finland the leaves of pale willow appear in May and are oval to egg-like and either have a fully serrated or shallowly sawn leaf edge. The leaf blade may be sparsely hairy or glabrous. The leaf tip is clearly visible and is often slightly crooked. Flowering occurs while coming into the leaf, and the female catkins become comparatively long when they reach the fruiting stage [52] (Figure 1A). On the whole, meadow willow gives a very vital impression; the foliage is rarely attacked by pests. Meadow willow occurs throughout Finland but not as commonly as many other willow species, and this was also reflected during our willow collections, since we found only one individual of this species. Overall, pale willow has a continental distribution and occurs especially in the taiga area of Siberia [15].

#### 3.1.2. Dark-Leaved Willow—*Salix myrsinifolia* (Salisb.), Voucher No. 856575

Dark-leaved willow is a shrub or small tree growing to a height of 2–5 m. Our specimen was a small male tree with a height of about 3 m, and the winter collection was performed in April 2022 (Figure 1C). The new annual shoots are greenish-brown and sparsely hairy. The buds are hairy-glabrous. The stipules are large, kidney-like or ovoid, and the edge of the stipules is provided with glands. An important characteristic is that the leaves turn black when they are dried. The leaf blade is sparsely hairy both on the underside and on the top, shiny on the top and bluish-green on the underside. Dark-leaved willow blooms shortly before the leaves emerge. The inflorescence (catkin) is short with two stamens per flower in male catkins and one pistil per flower in female catkins. The pistil is long-stalked and usually glabrous [52].

#### 3.1.3. Tea-Leaved Willow-*Salix phylicifolia* (L.), Voucher No. 856576

Tea-leaved willow is one of the most common willow species in Finland. It often grows like a shrub and usually becomes approximately 0.5–2 (−3) m high. The branches are upright, and the new annual shoots are shiny and red to greenish or yellowish brown. The leaves are lanceolate to oval and often lack stipules. The upper leaf surface is dark green and shiny, while the below-leaf surface is bluish green and slightly waxy. It blooms before the leaves have emerged. We have collected five specimens of tea-leaved willow from Lammi, which reflects the common occurrence of this willow species in Finland. Three of our collected specimens were male individuals and two were female. In this paper, we have used a male plant, which had reached a height of 4–5 m (Figure 1D). It was growing in Lammi on meadow-like vegetation near the roadside.

#### 3.1.4. Weeping Crack Willow-*S. x pendulina* (Wender.), Voucher No. H856569

*S. x pendulina* is a hybrid between *S. euxina* (crack willow) and *S. babylonica* (Babylon weeping willow). Sometimes it is considered that even *S. alba* (white willow) is included in the hybrid [53]. We collected dormant twigs in February 2022 from *S. x pendulina* from a stand of trees near Seurasaari (Helsinki). *S. x pendulina* has slender, green-brown and shiny branches. The leaves are narrow, lanceolate, shiny and almost hairless on the upper side, while the underside is bright green. The leaves thereby differ from those of the white willow (*S. alba*), which are densely silvery hairy on the upper side. The buds are narrow and bright green. The male and female flowers are on separate individuals, and we collected two female specimens, of which the other was used for our antibacterial screenings in this paper (Figure 1B). The female flowers are reduced to pistils with a catkin scale at the base, and they are borne on long catkins. *S. x pendulina* was brought to Finland from Russia in the 19th century [54].

### 3.2. Extraction Yields

**Figure 2:**
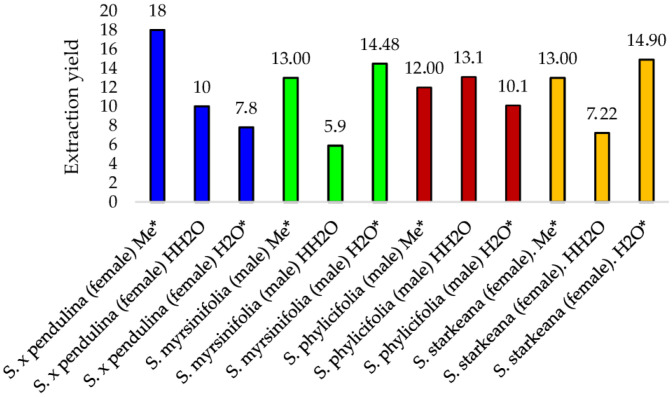
Extraction yields resulting from MeOH and water extraction of *Salix* species. Me*, cold methanol; H_2_O*, cold water extracts; HH_2_O, decoctions. Each species is marked with a separate color.

Extraction yields resulting from methanol, cold water and hot water extraction of *Salix* twig material are presented in Figure 2. The yields depend on the anatomical structure of the *Salix* twigs, such as the size and number of vessel elements, the porosity and fibrosity. Some *Salix* spp. are more fibrous than other species, for example; the twigs of *S. myrsinifolia* were more fibrous than those of the other *Salix* species, and thus, the total size of the extractable area is smaller for this species compared to the others. Methanol extraction resulted in the highest extraction yield of 18% for *S. x pendulina*, followed by *S. starkeana* and *S. myrsinifolia* (both giving a 13% extraction yield, Figure 2). Moreover, the amount of extractable material when using water extraction varied markedly from species to species and depending on the extraction temperature. For example, *S. myrsinifolia* and *S. starkeana* twigs contained a high concentration of compounds that dissolved in cold water but a smaller number of compounds in the decoctions, while for *S. x pendulina* and *S. phylicifolia*, the opposite was true. According to our UPLC-DAD data, the methanol extracts of the twigs of *S. myrsinifolia* contained a high number of procyanidins, as well as flavonoids and salicinoids (Table 1 and Table 2).

### 3.3. Antimicrobial Effects of Salix Extracts

Results of the growth-inhibitory effects of methanol and water extracts of the twigs of the *Salix* species are summarized in Table 1. Most of the *Salix* spp. showed mild growth inhibition against the bacteria, with MIC values between 625 and 2500 µg/mL, and many extracts did not reach the MIC when tested at 2500 µg/mL, showing a percentage growth inhibition below ninety percent.

**Figure 3:**
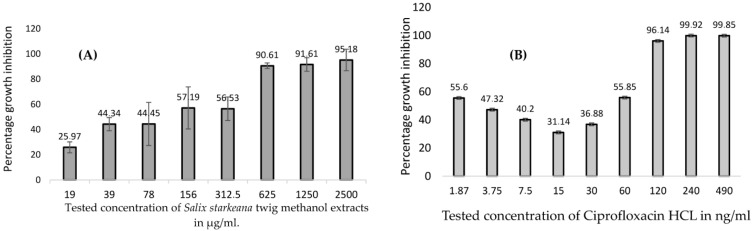
(**A**) Dose-response effects of a *Salix starkeana* twig methanol extract in µg/mL and (**B**) ciprofloxacin hydrochloride in ng/mL against *Bacillus cereus.* The results are expressed as the means of duplicates ± SEM.

Twig methanol extracts of *S. starkeana* and *S. myrsinifolia* were among the most growth inhibitory and were especially active against *B. cereus* (MIC 625 and 1250 µg/mL, respectively) and *S. aureus* (MIC 1250 µg/mL for both). Moreover, the growth-inhibitory effects of *S. starkeana* were dose-dependent against *B. cereus* (Figure 3A). However, only a methanol extract of the twigs of *S. myrsinifolia* was active against *E. coli*, resulting in 86% growth inhibition at 2500 µg/mL. Although a number of *Salix* extracts showed growth inhibition against *P. aeruginosa*, the MIC could not be reached when the extracts were screened at a concentration of 2500 µg/mL. For example, at 2500 µg/mL, a decoction of *S. myrsinifolia* and a methanol extract of *S. starkeana* inhibited 86 and 82 percent, respectively, of the growth of *P. aeruginosa*, and the results correlated well with large zones of inhibition (IZD 22 and 25 mm, respectively). Moreover, decoctions of *S. myrsinifolia* and *S. phylicifolia* and a methanol extract of *S. phylicifolia* were good inhibitors of the growth of *S. aureus*. Methanol extracts of *Salix x pendulina* resulted in good growth inhibition against *P. aeruginosa*, *B. cereus* and *S. aureus*, with inhibition zone diameters (IZDs) ranging from 22 to 25 mm. In addition, the agar diffusion result of the *S. x pendulina* extract against *P. aeruginosa* correlated well with the microplate method result (81% growth inhibition).

Ciprofloxacin hydrochloride showed a dose-dependent response with an MIC value at 60 ng/mL against *B. cereus* (Figure 3B). In addition, rifampicin was also found to be active against *B. cereus* with an MIC of 0.244 µg/mL (Table 1).

Interestingly, there was a large difference between the antibacterial activities of the hot- and cold-water extracts of *S. phylicifolia*, with the hot-water extract giving an IZD of 21 mm against *S. aureus*, whereas the cold-water extract showed just a small growth inhibitory effect against this bacterium. According to our previous experience, there can be large within-species variation between the antibacterial effects of hot- and cold-water extracts of *Combretum* and *Terminalia* species [41].

**Table 1:** Antibacterial effects of methanol and water extracts of *Salix* twigs and some pure compounds occurring in the *Salix* spp.

Plant Extracts	*P. aeruginosa*		*S. aureus*		*E. coli*		*Bacillus cereus*	
	IZD	MIC/GI%	IZD	MIC/GI%	IZD	MIC/GI%	IZD	MIC/GI%
*S. pendulina.* HH_2_O	NA	˃2500 (CI 44)	16.83 ± 0.4	NA	14.00 ± 0.0	NA	**21.67 ± 0.3**	˃2500 (CI 11)
*S. pendulina*. H_2_O*	NA	NA	16.00 ± 0.3	˃2500 (CI 44)	NA		NA	NA
*S. pendulina*. Me*	**25.00 ± 0.1**	**˃** **2500 (CI 80)**	**22.00 ± 0.4**	NT	NA		**22.00 ± 0.0**	˃2500 (CI 51)
*S. phylicifolia.* Me*	**26 ± 0.4**	˃2500 (CI 71)	**24.00 ± 0.2**	2500 * (CI 91 *)	NT	NT	**23.00 ± 0.2**	˃2500 (CI 58)
*S. phylicifolia*. HH_2_O	**20.5 ± 0.2**	˃2500 (CI 36)	**21.00 ± 0.2**	2500 * (CI 106 *)	NA		19.5 ± 0.3	59.29 (CI 59)
*S. phylicifolia*. H_2_O*	**23.00 ± 0.4**	˃2500 (CI 68)	NT	˃2500 (CI 52)	NA		15.4 ± 0.0	˃2500 (CI 31)
*S. myrsinifolia*. Me*	**21.33 ± 1.2**	˃2500 (CI 63)	**24.17 ± 0.4**	1250 * (CI 93 *)	**17.00 ± 0.0**	**˃** **2500 (CI 86)**	**23 ± 0.2**	1250 * (CI 99 *)
*S. myrsinifolia.* HH_2_O	**24 ± 0.3**	**˃** 2500 (CI 87 *)	**22.00 ± 0.3**	2500 * (CI 108 *)	20 ± 0.2	˃2500 (CI 56)	**22 ± 0.4**	2500 * (CI 106 *)
*S. myrsinifolia*. H_2_O*	**20 ± 0.3**	˃2500 (CI 51)	NT	**˃** 2500 (CI 83 *)	NA		**21 ± 0.0**	**˃** 2500 * (CI 89 *)
*S. starkeana.* Me*	**23 ± 0.0**	**˃** 2500 (CI 83 *)	**25 ± 0.3**	1250 * (CI 99 *)	NA		**24 ± 0.3**	625 * (CI 91 *)
*S. starkeana*. HH_2_O	**22.00 ± 0.3**	˃2500 (CI 57)	20.00 ± 0.4	˃2500 (CI 61)	NA		19.00 ± 0.0	**˃** 2500 (CI 87 *)
*S. starkeana.* H_2_O*	17 ± 0.4	˃2500 (CI 62)	16 ± 0.2	˃2500 (CI 21)	NA		18 ± 0.3	˃2500 (CI 40)
**Pure Compounds and Antibiotics**								
Procyanidin B1		˃500 (CI 49)		NT		NT		**250 (CI 95)**
Catechin		˃500 (CI 33)		NT		NT		**250 (CI 98)**
Naringenin		˃500 (CI 39)		NT		NT		NT
Tremulacin		˃500 (CI 47)		NT		NT		NT
Acetyl-salicortin		˃500 (CI 20)		NT		NT		˃500 (CI 76)
Salicortin		˃500 (CI 20)		NT		NT		˃500 (CI 68)
Salireposide		˃500 (CI 23)		NT		NT		NT
Populin		˃500 (CI 39)		NT		NT		NT
Salicyloyl salicin		˃500 (CI 39)		NT		NT		NT
Salicin		˃500 (CI 26)		NT		NT		NT
Taxifolin		˃500 (CI 33)		NT		NT		**250 (CI 99)**
*trans-m*-HCA		˃500 (CI 26)		NT		NT		˃500 (CI 70)
*trans-p*-HCA		˃500 (CI 26)		NT		NT		**250 (CI 99)**
Tetracycline		**62.5 (CI 100)**		**0.24 (CI 94)**		**0.98**		**0.49 (CI 100)**
Ciprofloxacin HCL		NT		NT		NT		**0.06 (CI 96)**
Rifampicin		NT		**0.015 (CI 99)**		**15.63**		**0.244 (CI 96)**

For the agar diffusion test, the extracts were tested at 10 mg/mL. For the microplate method, a starting concentration of 2500 µg/mL was used for the extracts and 500 µg/mL for the pure compounds. The agar diffusion results were calculated as the mean of triplicates ± SEM. The percentage growth inhibition results (% GI) were calculated as the mean of duplicates ± SEM. HH_2_O, decoction; H_2_O*, maceration; Me*, methanol extract; MIC, minimum inhibition concentration in µg/mL; IZD, inhibition zone diameter in mm; GI %, percentage of growth inhibition indicated as IC; IC, inhibitory concentration (percentage growth inhibition at the indicated concentration); NA, not active; NT, not tested; HCA, hydroxycinnamic acid. The underlined bold results marked with asterisks indicate the best results against the bacterial growth showing growth inhibition of 90% or more.

### 3.4. Time-Kill Effects of an S. myrsinifolia Twig Methanol Extract against B. cereus

**Figure 4:**
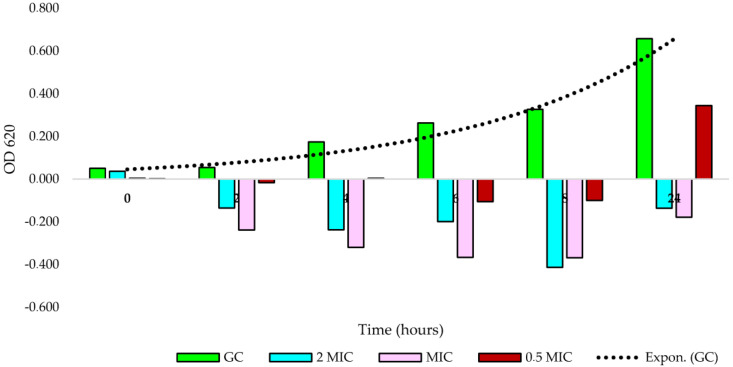
Time-kill assay result from an analysis measuring changes in optical density at 620 nm resulting from the growth inhibition of a *Salix myrsinifolia* twig methanol extract on *B. cereus* during an incubation time of 24 h. Light-blue bars indicate a concentration of 2 × MIC (2.5 mg/mL), blue bars the MIC (1.25 mg/mL) and red bars the 0.5 × MIC (625 µg/mL). GC, growth control.

Since a methanol extract of *S. myrsinifolia* twigs was among the most active extracts in inhibiting the growth of *B. cereus*, a closer analysis was performed to investigate the dynamic effects of this extract on the growth of *B. cereus* during a 24 h incubation time. We noticed that at all tested concentrations, 2 × MIC (2.5 mg/mL), MIC (1.25 mg/mL) and 0.5 × MIC (625 µg/mL), there was strong inhibition of *B. cereus* growth, as measured using the OD620 (Figure 4). Moreover, no growth at all was observed for the 2 × MIC and MIC concentrations, whereas at the 0.5 × MIC concentration (625 µg/mL), there was slight bacterial growth after 24 h (Figure 4).

**Figure 5:**
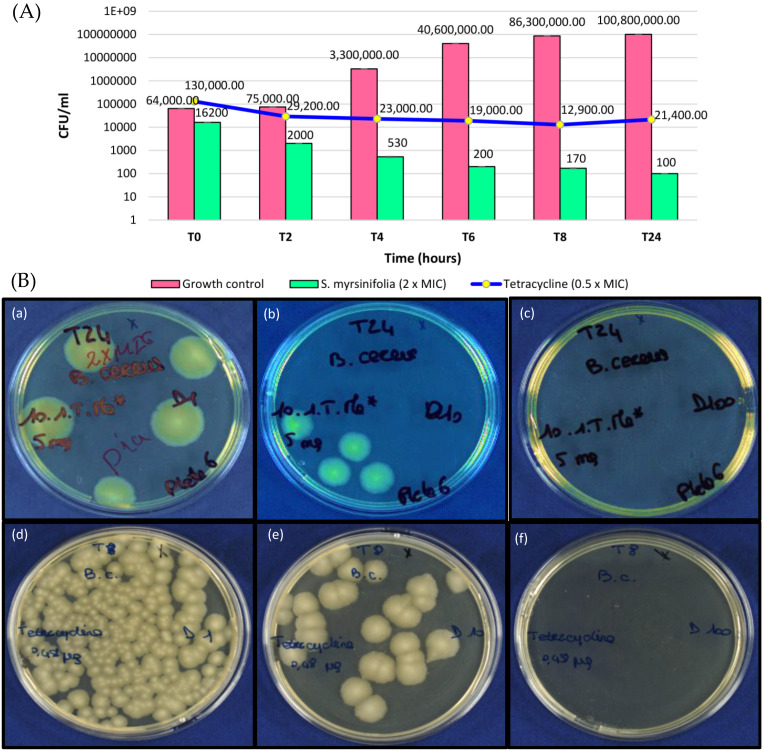
(**A**) Time-kill effects of a *S. myrsinifolia* methanol extract of the twigs and tetracycline against *B. cereus* using a plate count method. The plant extract (green bar) was tested at its 2 × MIC concentration (5000 µg/mL) and tetracycline at 0.5 × MIC (0.244 µg/mL). The resulting colonies were counted during every second hour over 24 h of incubation. The results are expressed as the mean CFU/mL of duplicates ± SEM. (**B**) Time-kill effects of a *S. myrsinifolia* methanol twig extract at 2500 µg/mL (**a**–**c**) and tetracycline at 0.24 µg/mL (**d**–**f**) against *B. cereus* as illustrated by the plate count method. All Petri dishes shown in this figure were recorded after an incubation time of 24 h. For the test, various dilutions, including (**a**) 1:10, (**b**) 1:100 and (**c**) 1:1000, were used in order to make it possible to count the colonies. (**d**–**f**) Show the dilutions of tetracycline used for the plate count (1:10, 1:100 and 1:1000). The numbers of CFU/mL were counted only for those dilutions containing a countable number of colonies (<300 colonies per dish).

To confirm the results obtained for time-kill using OD620 measurements, plate counts were used (Figure 5A,B). Results of the time-kill effects of the methanol extract of *S. myrsinifolia* against *B. cereus* using the plate count method are shown in Figure 5A. At a concentration of 2 × MIC (5000 µg/mL), the extract was more effective than tetracycline (tested at its 0.5 × MIC concentration, 0.24 µg/mL). Moreover, the extract was inhibiting bacterial growth throughout the incubation of 24 h and resulted in a colony count of 100 CFU/mL after 24 h compared to 21,400 CFU/mL for tetracycline. Thus, it can be concluded that the *S. myrsinifolia* methanol twig extract at the 2 × MIC concentration of 5 mg/mL has a bactericidal effect on the growth of *B. cereus*.

### 3.5. Antibacterial Effects of Compounds Present in Salix spp.

A number of condensed tannins and their monomers, including procyanidin B1 and catechin, as well as many flavonoids and phenolic acids, are present in the investigated *Salix* species according to our LC-MS results. Thus, the growth-inhibitory effects of some *Salix* pure compounds were tested against *P. aeruginosa* and *B. cereus*, and the results are presented in Table 1. Altogether, as expected the pure compounds were more effective against *B. cereus* than against *P. aeruginosa*.

The condensed tannin, procyanidin B1 and its monomer, catechin, showed a dose- dependent growth-inhibitory response and a good effect against *B. cereus* with an MIC value of 250 µg/mL (percentage growth inhibition, GI, of 95% and 98%, respectively). Thus, procyanidin B1 and catechin were 2.5 and 5 times more effective, respectively, than the most effective extracts; e.g., a methanol extract of *S. starkeana* (MIC 625 µg/mL) and a methanol extract of *S. myrsinifolia* (MIC 1250 µg/mL). Likewise, the flavonoid taxifolin and *trans-p*-hydroxycinnamic acid (syn. *trans-p*-coumaric acid or *trans*-4-coumaric) presented good growth-inhibitory effects at 250 µg/mL (99% GI) against *B. cereus*. Interestingly, *trans-m*-hydroxycinnamic acid (syn. *trans-m*-coumaric acid or *trans*-3-coumaric) (MIC > 500 µg/mL) was not as active as *trans-p*-hydroxycinnamic acid (MIC 250 µg/mL) against *B. cereus*.

At 500 µg/mL, procyanidin-B and the salicylate tremulacin inhibited more than 49% and 48%, respectively, of the growth of *P. aeruginosa* (Table 1). The rest of the compounds resulted in 19.9–39.5% growth inhibition of *P. aeruginosa* when tested at 500 µg/mL, with salicyloyl salicin, populin and naringenin being the most active compounds and acetylsalicortin the least active compound.

### 3.6. Antioxidant Effects of a Methanol Twig Extract of S. starkeana

**Figure 6:**
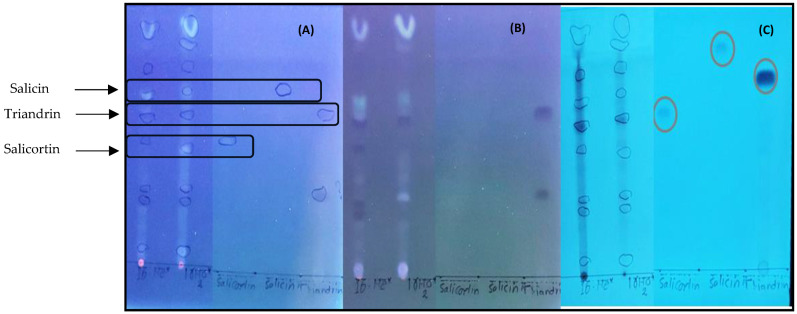
Thin-layer chromatography combined with antioxidant analysis of a methanol extract and water maceration from the twigs of *S. starkeana*. Extracts and standards (**A**) after spraying with the 2,2-diphenoyl-1-picrylhydrazyl (DPPH) reagent, with antioxidant compounds as yellow spots, (**B**) at 366 nm and (**C**) at 245 nm.

Our qualitative RP-18 TLC analysis revealed a number of components in the *S. starkeana* water maceration and methanol extracts. Altogether, nine spots were visible on the TLC plates at 254 and 366 nm (Figure 6). Many of these compounds showed strong antioxidative effects, as they turned intensively light yellow after spraying with the DPPH reagent. Triandrin, salicortin and salicin were used as standard compounds, of which salicin and salicortin seemed to show the strongest antioxidant effect (Figure 6). Moreover, some very polar spots at the top of the TLC chromatogram, which could correspond to procyanidins (present in Table 2 at Rt 0.5 min), show strong antioxidant effects.

### 3.7. Phytochemical Analysis of a S. myrsinifolia Methanol Twig Extract Using UPLC/QTOF-MS

**Figure 7:**
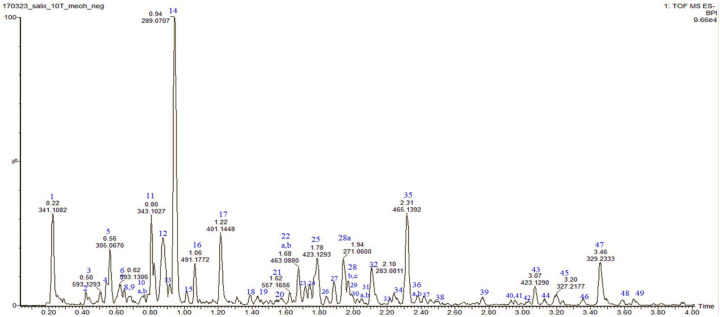
Total ion chromatogram (TIC) (in negative ion mode) and the full-scan mass spectra of the major metabolites detected in a *Salix myrsinifola* twig methanol extract. The scan spectra were obtained via UPLC/QTOF-MS in negative ion mode. Compound numbers and compound numbers including letters are as in Table 2 and in the text.

**Figure 8:**
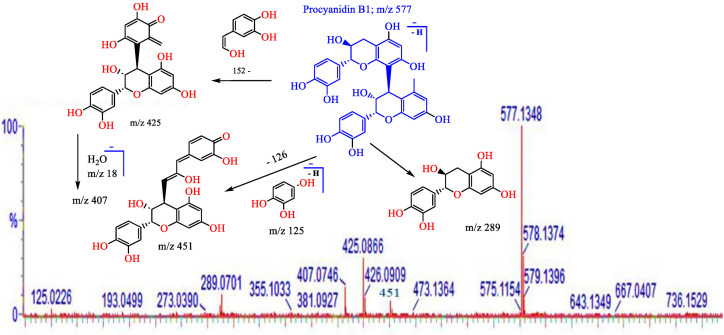
Mass spectrum and fragmentation pattern of procyanidin B1 C_30_H_26_O_12_; calculated mass, *m*/*z* 577.1346; measured mass [M–H]^−^, 577.1348.

**Figure 9:**
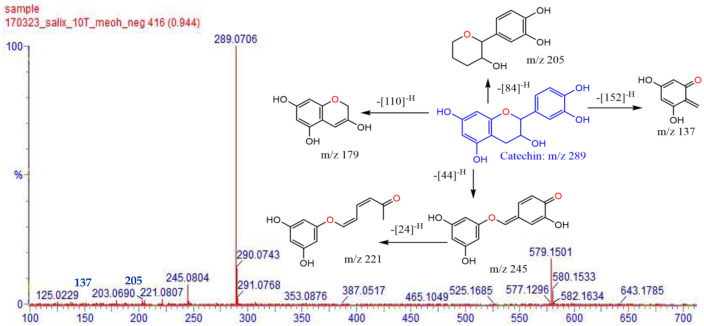
Mass spectrum and fragmentation pattern of catechin (flavan-3-ol); C_15_H_14_O_6_; calculated mass, *m*/*z* 289.0706; Measured mass [M–H]^−^, 289.0705.

**Figure 10:**
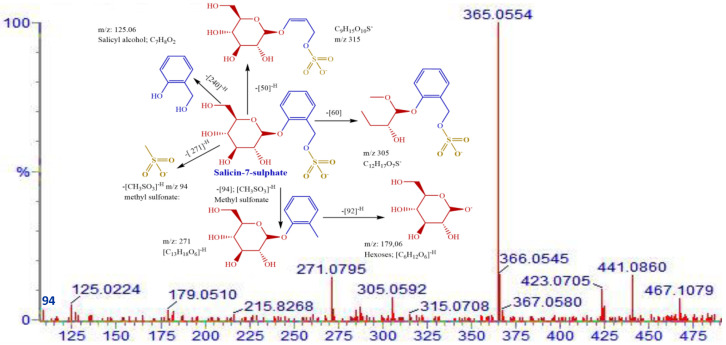
Mass spectrum and fragmentation pattern of salicin-7-sulfate; C_13_H_18_O_10_S); calculated mass, *m*/*z* 366.0542; measured mass, [M–H]^−^ 365.0544.

**Figure 11:**
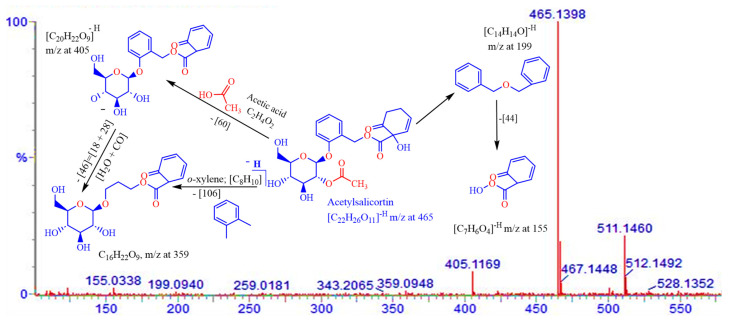
Spectra and fragmentation pattern of acetylsalicortin; (C_22_H_26_O_11_)^−^ calculated mass, *m*/*z* 465.1397; measured mass, [M–H] 465.1394.

A methanol extract of the twigs of *S. myrsinifolia* was chosen for a phytochemical analysis using UPLC/QTOF-MS due to its good antibacterial potential. Altogether forty-nine compounds were tentatively characterized, of which most are polyphenols, including 13 compounds related to the procyanidins or their flavan-3-ol monomers, catechin, epicatechin and epicatechin gallate (Figure 7, Table 2). The mass spectra and the MS/MS fragmentation pattern of procyanidin B1, acetylsalicortin, salicin-7-sulphate, benzyl ß-primeveroside and catechin in the *S. myrsinifolia* extract were elucidated and are presented in Figure 7, Figure 8, Figure 9, Figure 10 and Figure 11. The mass spectra of the compounds were compared with corresponding standard compounds (data shown as Supplementary Materials).

Several of the detected molecules in the twig methanol extract of *S. myrsinifolia* are condensed tannins or proanthocyanidins (Table 2). Proanthocyanidins or condensed tannins include oligomers and polymers based on flavan-3-ol molecules, such as catechin, epicatechin and gallocatechin. In the B-type procyanidins (PCs), the flavan monomers are linked to each other mainly by C4–C8 bonds and sometimes via C4–C6 bonds [55,56,57,58,59,60]. Compounds **3** and **6** with a molecular ion [M–H]^−^ at *m*/*z* 593 comprise catechin-gallocatechin units. Catechin-gallocatechin procyanidin isomers with a molecular ion at *m*/*z* 593 were previously found in the leaves of *Salix alba* [61] and in pomegranate (*Punica granatum*) peel [62]. In addition, at Rt 0.88 min, procyanidin B1 (PA B1) was present (compound **12**), showing a molecular ion [M–H]^−^ at *m*/*z* 577.1348 (Table 2). Procyanidin B1 is a dimer of (−)-epicatechin and (+)-catechin.

Figure 8 shows the mass spectrum and fragmentation pattern of procyanidin B1 as obtained via LC-MS/MS. The fragmentation pattern of the PA B1 in *S. myrsinifolia* was in accordance with data shown by Enomoto and co-workers [60]. Cleavage of the interflavan bond between the catechin and epicatechin monomers of procyanidin B1 yielded a fragment of catechin at *m*/*z* 289 (Quinone-methide, QM cleavage) (Figure 8), whereas the cleavage a hydroxy-vinyl-benzene-diol ion at *m*/*z* 152 from the deprotonated molecular ion at *m*/*z* 577 of PA B1 resulted in a fragment at *m*/*z* 425 [M–H-152]^−^, corresponding to the retro Diels-Alder reaction [58,60,63]. Moreover, elimination of a molecule of water at *m*/*z* 18 (H_2_O from [M–H]^−^ *m*/*z* 425) resulted in a fragment at *m*/*z* 407. In addition, we noticed the loss of a phloroglucinol group at *m*/*z* 126 (indicated as the deprotonated form at *m*/*z* 125 in Figure 7), generating a fragment of [M–H−451]^−^, which is a typical fragment of the procyanidin type B obtained via heterocyclic ring fission (HRF) [60,63,64].

Due to good signaling intensity and fragmentation accuracy (mass error −0.7 mDa), we detected catechin (compound **14**, Rt 0.94 min) as a major metabolite in *Salix myrsinifolia* twigs. The fragmentation pattern of catechin is shown in Figure 9. Loss of a CO_2_ unit [M–H–CO_2_]^−^ at the C ring of the flavanol skeleton [M–H-44]^−^ produces a product ion at *m*/*z* 245, a fragment typically observed in the MS/MS spectra of flavanols [65,66]. Furthermore, the most diagnostic fragments of a flavanol aglycone, such as catechin, were present due to the cleavage of the C ring [M–H-152]^−^ resulting in the ion fragments at *m*/*z* 137, *m*/*z* 125 (phloroglucinol) and *m*/*z* 179. These fragments could also be formed through retro-Diels–Alder (RDA) reactions due to the presence of four hydroxyl groups in the A- and B- rings, as well as to the fragment loss of [M–H-152]^−^ [64,67]. This information together with the product ion at *m*/*z* 289 [M–H]^−^ revealed the identity of compound **14** as the catechin ion. However, as shown in Figure 9, we also observed the occurrence of catechin dimers that are formed via oxidation due to a single C-O-C- bond. These dimers show an [2M-H]^−^ ion at *m*/*z* 579, which is further deprotonated at the cleavage linkage C-O-C giving a product ion spectrum of *m*/*z* 291, which is specific for C-O-C fragmentation and for oxidized dimeric catechins [68]. The presence of the ion at [M–H]^−^ *m*/*z* 577 (Figure 9) suggests the formation of an additional linkage between the two catechin units [69]. The difference between the catechin enantiomers, (+)-catechin and (-)-catechin is due to the stereochemical configuration of the hydroxyl- and hydrogen bonds at position C_2_, C_3_ and C_4_.

In addition, we detected salicin-7-sulfate (**8**)**,** a new core structure of a salicinoid (a phenolic glucoside). The fragmentation pattern of salicin-7-sulfate is shown in Figure 10. Salicin-7-sulfate (**8**) shows two main fragments, a salicyl alcohol fragment [C_7_H_8_O_2_] at *m*/*z* 125 and a methyl sulfonate moiety [CH_3_SO_3_]^−^ at *m*/*z* 94. The accurate mass of salicin-7-sulfate is *m*/*z* 365.0542 [M–H]^−^, with a chemical formula of C_13_H_18_O_10_S (calculated [M–H]^−^ 365.0554; *m*/*z* mass error −1.1 ppm and mDa −0.4) (Table 2). A fragment at *m*/*z* 271 (C_13_H_18_O_6_), resulting from the cleavage of the sulfonate moiety (*m*/*z* 94) from salicin-7-sulfate, is visible in the mass spectrum. Further deprotonation occurred at the glycone moiety from [M–H]−271]^−^ and corresponds to the cleavage of the hexose unit of glucose at *m*/*z* 179 [70,71]. The fragment loss of 271 and the absence of the fragment at *m*/*z* 259 indicates that the glucose moiety was connected to the benzene ring and not to the functional group at C_5_′ or to the methyl sulfonate moiety at *m*/*z* 94 [20,72].

We also detected the presence of the phenolic glucoside salicortin C_20_H_24_O_10_ (**25**) in the twig methanol extract of *S. myrsinifolia* at the ion chromatogram retention time Rt 1.79 min and showing an [M–H]^−^ ion at *m*/*z* 423.1288. One major fragment was observed at *m*/*z* 469, corresponding to the loss of *m*/*z* 46 of the CO and H_2_O from the molecular ion [M–H]^−^ (18 + 28)].

Compound (**35**) with an Rt at 2.31 min and the molecular formula C_22_H_26_O_11_ produced a deprotonated molecular ion at [M–H]^−^ 465.1402 (Figure 11). The peak was deacetylated [M–H–CH_3_CO] by the loss of an *m*/*z* 60 unit from the molecular ion, resulting in a fragment at *m*/*z* 405, giving an intense peak in the mass spectrum. Further fragmentation of a H_2_O + CO unit (*m*/*z* 46) from the *m*/*z* fragment 405 resulted in a fragment at *m*/*z* 359 C_16_H_22_O_9_. The fragmentation pattern of compound (**35**) was in agreement with the fragmentation pattern that we noticed for the corresponding standard compound of acetylsalicortin. Thus, compound (**35**) was assigned as acetylsalicortin.

We identified one compound belonging to the class of hydroxycinnamic acids (HCAs); compound (**11**) at Rt 0.79 min showed an [M–H]^−^ ion at *m*/*z* 341.0872 and is a caffeoyl isomer (C_15_H_18_O_9_). This compound is a phenolic steroid ester. In addition, protocatechuic acid-salicylhexoside (**27**) was present at 1.89 min showing an [M–H]^−^ ion at *m*/*z* 435.0917.

**Figure 12:**
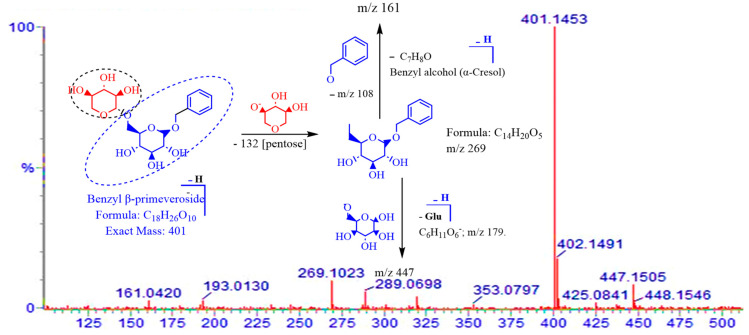
Spectra and fragmentation pattern of benzyl β-primeveroside; (C_18_H_26_O_10_)^−^ calculated mass, *m*/*z* 401,1448; measured mass, [M–H]^−^ 401,1452.

An *O*-glycosyl compound (compound **17**) containing benzyl alcohol connected with a sugar group through an *O*-glycosidic bond was detected at [M–H]^−^ *m*/*z* 401 and a retention time of 1.22 min. Loss of a pentose sugar (*m*/*z* 132) from the molecular ion at *m*/*z* 401.1456 ([M–H]^−^-132) is visible as a fragment (C_14_H_20_O_5_) at *m*/*z* 269 in the mass spectrum (Figure 12). Further fragmentation of the ion at *m*/*z* 269 (C_14_H_20_O_5_) produces a unit with the chemical formula C_7_H_8_O at *m*/*z* 108 [M–pentose–C_14_H_20_O_5_]^−^ and a hexose molecule [C_6_H_11_O_6_]^−^ at *m*/*z* 179. Therefore, compound (**17**) was assigned as benzyl-β-primeveroside (C_18_H_26_O_10_). Other flavonoids were identified based on a comparison to their corresponding standard compounds and were identified as quercetin glucoside (**22a**), taxifolin (**22b**), isorhamnetin glycoside (**30**), naringenin glucoside (**26**), naringenin (**28a**) and luteolin (**38**).

**Table 2:** Qualitative phytochemical analysis of a *Salix myrsinifolia* twig methanol extract. Molecular masses were obtained in negative ionization mode and the mass measurement error is described as the molecular mass (mDa) error and the mass error (ppm).

Compounds	Rt UPLC	*m*/*z* [M–H]^−^	Chem. Formula	Calc *m*/*z*	Error (mDa)	Error (ppm)
Caffeoylhexose (1)	0.22	341.0872	C_15_H_18_O_9_	341.1079	0.2	0.6
Salicylate derivative (2)	0.42	411.0237	C_5_H_12_N_6_O_16_	411.0232	0.5	1.2
**Procyanidin (catechin-gallocatechin dimer) (3)**	0.50	**593.1290**	**C_30_H_26_O_13_**	**593.1295**	−0.5	−0.8
Protocatechuoylglucose I (4)	0.54	315.0714	C_13_H_16_O_9_	315.0716	−0.2	−0.6
**Epigallocatechin (5)**	0.56	**305.0661**	C_15_H_14_O_7_	305.0661	0.0	0.0
**Prodelphinidin (catechin-gallocatechin dimer) (6)**	0.62	**593.1294**	**C_30_H_26_O_13_**	**593.1295**	−0.1	−0.2
Protocatechuoylglucose II (7)	0.64	315.0708	C_13_H_16_O_9_	315.0716	−0.8	−2.5
**Salicin-7-sulfate (8)**	**0.68**	**365.0544**	**C_13_H_18_O_10_S**	**366.0542**	**−0.4**	**−1.1**
**Epigallocatechin-epicatechin-epicatechin (9)**	**0.75**	**881.1914**	**C_45_H_38_O_19_**	**881.1929**	**−1.5**	**−1.7**
Salicin (10a) *	**0.76**	**285.0977**	**C_13_H_18_O_7_**	**285.0977**	**0.0**	**0.0**
Tryptophan (10b)	0.78	203.0814	C_11_H_12_N_2_O_2_	203.0821	−0.7	−3.4
**Caffeoylhexose isomer** (11)	0.79	341.0872	C_15_H_18_O_9_	341.0873	−0.1	−0.3
Procyanidin B1 (12) *	**0.88**	**577.1348**	**C_30_H_26_O_12_**	**577.1346**	**−0.2**	**−0.3**
**Salicylic acid glucoside (13)**	**0.92**	**299.0752**	**C_13_H_16_O_8_**	**299.0767**	**−1.5**	**−5.0**
**Catechin (14)**	**0.94**	**289.0706**	**C_15_H_14_O_6_**	**289.0712**	**−0.7**	**−2.4**
**Coumaric acid 2-glucoside** (15)	1.01	325.0922	C_15_H_18_O_8_	325.0923	−0.1	−0.3
Flavonoid pentose (16)	1.06	491.1765	C_21_H_32_O_13_	491.1765	0.0	0.0
**Benzyl-β-primeveroside (17)**	1.21	401.1452	C_18_H_26_O_10_	401.1448	0.4	1.0
Unknown compound	1.31	357.0813	C_15_H_18_O_10_	357.0822	−0.9	−2.5
**Procyanidin B1 dimer (18)**	1.39	577.1322	C_30_H_26_O_12_	577.1346	−2.4	−4.2
**Salicin derivative (19a)**	1.40	373.1183	C_16_H_22_O_10_	373.1135	4.8	
Catechin dervative (19b)	1.45	479.0815	C_21_H_20_O_13_	479.0826	−1.1	−2.3
Unknown compound (**20**)	1.57	715.1660	C_37_H_32_O_15_	715.1663	−0.3	−0.4
Robinetidinol-6-Catechin (21a)	1.62	557.1644	C_28_H_30_O_12_	557.1659	−1.5	−2.7
Rutin syn. quercetin rutinoside (21b)	1.65	609.1445	C_27_H_30_O_16_	609.1456	−1.1	−1.8
Quercetin-3-O-glucoside (Isoquercitrin) (22a)	1.68	463.0872	C_21_H_20_O_12_	463.0877	−0.5	−1.1
**Taxifolin (22b)**	**1.69**	**303.0500**	**C_15_H_12_O_7_**	**303.0505**	**−0.5**	**−1.6**
Epicatechin (23)	1.74	289.0707	C_15_H1_4_O_6_	289.0712	−0.5	−1.7
Luteolin glucoside (24)	1.76	447.0925	C_21_H_20_O_11_	447.0927	−0.2	−0.4
Salicortin (25) *	**1.79**	**423.1288**	C_20_H_24_O_10_	423.1291	−0.3	−0.7
Naringenin glucoside (26)	1.84	**433.0762**	C_20_H_18_O_11_	433.0771	−0.9	−2.1
**Protocatechuic acid-salicylhexoside (27)**	1.89	435.0917	C_20_H_20_O_11_	435.0927	−1.0	−2.3
Catechin derivative (28a)	**1.94**	427.1019	C_22_H_20_O_9_	427.1029	−1.0	−2.3
**Naringenin (28a)**	1.94	**271.0599**	C_15_H_12_O_5_	271.0606	−0.7	−2.6
Unknown compound (**28b**)	1.94	855.2122	C_44_H_40_O_18_	855.2136	−1.4	−1.6
Catechin derivative (28c)	1.94	289.0701	C_15_H_14_O_6_	289.0712	−1.1	−3.8
Unknown compound (**29**)	1.95	439.1603	C_21_H_28_O_10_	439.1604	−0.1	−0.2
**Isorhamnetin glycoside (30)**	**2.00**	**477.1024**	C_22_H_22_O_12_	477.1033	−0.9	−1.9
Naringenin derivative (31a)	2.02	271.0599	C_15_H_12_O_5_	271.0606	−0.7	−2.6
Luteolin derivative (31b)	2.03	287.0545	C_15_H_12_O_6_	287.0556	−1.1	−3.8
**Helicin (salicylaldehyde-B-D-glucoside) (32)**	2.10	283.0808	C_13_H_16_O_7_	283.0818	−1.0	−3.5
**Salicortin derivative (33)**	2.25	423.1646	C_21_H_28_O_9_	423.1655	−0.9	−2.1
Acetyl-salicortin with formic acid (34)	2.31	511.1441	C_23_H_28_O_13_	511.1452	−1.1	−2.2
**Acetyl-O-salicortin (35)**	**2.32**	**465.1394**	C_22_H_26_O_11_	465.1397	−0.3	−0.6
**Salireposide (36a)**	2.32	**405.1174**	C_20_H_22_O_9_	405.1186	−1.2	−3.0
Catechin pentoside (36b)	2.37	421.1288	C_20_H_22_O_10_	421.1287	0.1	0.2
**Salicortin derivative (37)**	2.40	423.1646	C_21_H_28_O_9_	423.1655	−0.9	−2.1
**Luteolin (38)**	**2.54**	**285.0391**	**C_15_H_10_O_6_**	**285.0399**	**−0.8**	**−2.8**
Unknown compound (**39**)	2.78	585.1600	C_29_H_28_O_13_	585.1608	−0.8	−1.4
Naringenin derivative (40)	2.88	271.0602	C_15_H_12_O_5_	271.0606	−0.4	−1.5
Unknown compound (**41**)	2.95	581.2022	C_38_H_30_O_6_	581.1964	5.8	
**Acetyl-salicortin derivative (42)**	3.04	465.1387	C_22_H_26_O_11_	465.1397	−1.0	−2.1
**Salicortin derivative (43)**	3.07	423.1289	C_20_H_24_O_10_	423.1291	−0.2	−0.5
Unknown compound (**44**)	3.12	809.3012	C_42_H_50_O_16_	809.3021	−0.9	−1.1
Unknown compound (**45**)	3.20	327.2172	C_18_H_32_O_5_	327.2171	0.1	0.3
**Salicortin derivative (46)**	3.36	423.1291	C_20_H_24_O_10_	423.1291	0.0	0.0
Unknown compound (**47**)	3.46	329.2322	C_18_H_34_O_5_	329.2328	−0.6	−1.8
Unknown compound (**48**)	3.58	703.0000	C_45_H_35_O_8_	703.0000	0.0	0.0
Methylated catechin glucopyranoside (49)	3.65	465.1389	C_22_H_26_O_11_	465.1397	−0.8	−1.7

Mass error was explained in parts per million (ppm) and in mass Dalton (mDa); * Mass and UV spectra for the standard compounds are shown as Supplementary Materials; The bold text indicates the most important compounds that were identified and discussed in this article

## 4. Discussion

### 4.1. Antimicrobial Effects of Salix Extracts in Relation to Other Studies

Our study indicates that polar twig extracts of *Salix myrsinifolia*, *S. starkeana*, *S. phylicifolia* and *S. x pendulina* have good antibacterial potential with MIC values ranging from 625 to 2500 µg/mL. Moreover, the growth-inhibitory effects were most prominent against the Gram-positive bacteria, *S. aureus* and *B. cereus*, although some extracts also showed good growth inhibition against *P. aeruginosa*. However, none of the tested extracts were active against *Candida albicans* and *E. coli*.

Among our screened species, *S. starkeana* was not tested previously for its growth-inhibitory effects against human pathogenic bacteria. We found that a methanol extract of *S. starkeana* was the most effective of all our screened extracts against *B. cereus* with an MIC value of 625 µg/mL. Our result is in line with other studies of the antibacterial effects of *Salix* spp. extracts against *Bacillus* spp. For example, a methanol extract of the aerial parts of *S. babylonica* inhibited the growth of *B. subtilis* [73].

We found that methanol extracts and a decoction of the twigs of *S. myrsinifolia* inhibit the growth of *P. aeruginosa*, *B. cereus* and *S. aureus*, with MIC values from 1250 to >2500 µg/mL. Interestingly, at 2500 µg/mL, a decoction of *S. myrsinifolia* showed 86.88% growth inhibition against *P. aeruginosa*. The methanol extract of the twigs of *S. myrsinifolia* was the second most active of the *Salix* extracts against *B. cereus*, showing an MIC of 1250 µg/mL, and when this extract was tested at its 2 × MIC concentration (5000 µg/mL) for its growth kinetic effects, it showed a bactericidal effect against *B. cereus* during a 24 h period of growth and resulted in fewer CFU/mL than tetracycline at all timepoints of the assay (Figure 5A). Our results agree with a study of Mai et al. [74], which indicates that methanol extracts of *S. babylonica* leaves at a dose of 2 × MIC (6 and 4 mg/mL, respectively) were effective in reducing the growth of *Vibrio alginolyticus* and *V. parahaemolyticus* during a 24 h period of growth. However, we did not find any growth-inhibitory activity for the methanol and water extracts of *S. myrsinifolia* against *E. coli*. An earlier study showed that *Salix myrsinifolia* chloroform extracts of the aerial shoots display antibacterial effects against *B. subtilis*, *P. aeruginosa*, *S. aureus* and *E. coli* with the diameters of inhibition zones ranging from 20 to 34 mm [38]. These findings are in line with our results, although they are not directly comparable to the antibacterial effects of our polar methanol and water extracts since chloroform extracts differ from these polar extracts in their chemical composition. Moreover, in agreement with our results, Tienaho et al. [75] reported good antibacterial effects of the hot-water bark extracts of clones of *S. myrsinifolia* against *S. aureus*. However, Tienaho et al. [75] also found that the hot water extracts inhibited the growth of *E. coli*, and this result might be due to them using a different extraction technique, involving an extraction reactor and a temperature of 80 °C during the 60 min of extraction, compared to our boiling of the extract for 5 min, which might not be enough to extract more lipophilic compounds.

Among the few papers that were published on the antimicrobial potential of *S. phylicifolia*, Tienaho et al. [75] reported that hot-water extracts of the bark were antibacterial against *S. aureus* and *E. coli*. The result is in line with our findings that methanol and a hot-water extract of *S. phylicifolia* effectively inhibit the growth of *S. aureus*. However, we found that the hot- and cold-water and the methanol extracts of *S. phylicifolia* did not inhibit the growth of *E. coli*.

Corresponding to our finding that methanol extracts of *Salix x pendulina* twigs inhibit *S. aureus* and *B. cereus*, González-Alamilla et al. [73] and Rangel-López et al. [76] also reported that the methanol extracts of the stem bark and leaves of *Salix babylonica*, a species closely related to *Salix x pendulina*, showed an MIC of 12.5 mg/mL and 25 mg/mL, respectively, against *B. subtilis*, *S. aureus* and *Streptococcus iniae*.

It has been stated that the total antimicrobial activity of a plant extract depends on the extraction yield and the MIC and should be calculated as the percentage yield of one gram of the extracted plant material divided to the minimum inhibitory concentration of the extract [59,77]. Thus, the highest total antimicrobial activity was shown using the methanol extracts of the *Salix* species in this present study, and especially based on the methanol extract of *S. starkeana* against *B. cereus.*

### 4.2. Antibacterial Potential of Pure Compounds Present in Salix Species

Our UPLC/QTOF-MS results show that a methanol extract of *S. myrsinifolia* twigs with good antibacterial properties contains a wide range of phenolic compounds. The antibacterial activity of the *Salix myrsinifolia* extract could be due to the presence of procyanidin B1 (**12**) and its dimer derivatives, such as compound (**18**), since our commercial procyanidin B1 compound gave an MIC of 250 µg/mL against *B. cereus*. Indeed, procyanidins have been found to possess good antibacterial activity. A high hydroxylation level in the B ring of the catechin monomer units of B--type procyanidins, in addition to a high hydroxylation level in the B ring of the catechin subunits in procyanidin dimers, trimers, tetramers and oligomers, increases the specific polarity (Figure 8) and thus the antimicrobial activity of procyanidin molecules [55,78]. Also, procyanidins were found to be responsible for cell wall disintegration in *S. aureus* [79]. Moreover, and interestingly, it has been found that procyanidins (PAs) up to trimer polymerization are bioavailable in plasma and urine after the oral feeding of procyanidin-rich extracts. The procyanidin dimer-B_2_ is rapidly absorbed into human plasma [80].

We found that (+)-catechin was one of the most abundant compounds in *S. myrsinifolia* twigs in accordance with Nissinen et al. [81]. Significantly, our commercial standard, catechin, inhibited the growth of *B. cereus* at an MIC of 250 µg/mL. Catechin (**14**) and its isomers (**9**) were found to inhibit the growth and biofilm formation of methicillin-resistant *S. aureus* (MRSA) [82], and catechin suppressed the oxidation caused by *P. aeruginosa* [83]. Both procyanidin and catechin were found earlier in *Salix sieboldiana* bark acetone extracts [84], in *Salix tetrasperma* bark methanol extracts [39] and in *Salix sachalinensis* bark hot-water extracts [51]. In addition, a comprehensive study on the proanthocyanidins in *S. alba* revealed a high number of procyanidin dimers, trimers and tetramers [61].

In our study, salicin (**10a**), as a pure compound, resulted in a low growth-inhibition percentage (%) against *P. aeruginosa*. Corresponding to our results, a previous study by Dou et al. [35] also showed that salicin has no activity against *S. aureus* at concentrations of 3 mg/mL. However, in another study, salicin isolated from the bark of *Salix tetrasperma* gave a 7 mm diameter of the inhibition zone (IZD) against *S. aureus* and *E. coli* at a concentration of 2500 µg/mL [85]. In addition, salicin was recently reported to have a DNA-mutation effect [35]. Moreover, salicin might play a crucial pharmacological role as a pro-drug agent when converted to an active molecule, such as saligenin (salicyl alcohol), via gut microbiota [86].

In this present study, we detected salicin-7-sulfate (**8**) in the twigs of *S. myrsinifolia*. To the best of our knowledge, this sulfated salicinoid has not been found previously in *S. myrsinifolia*. Salicin-7-sulfate is a novel salicinoid in *Salix* spp. that was previously found in the dormant winter stem material of the Korean species *Salix koriyanagi*, which is included in the UK National willow collection [20]. However, sulfated salicinoids were found in *Populus* spp., a genus that is closely related to *Salix* [71]. The sulfur group in salicin-7-sulfate (**8**) might contribute to the antibacterial effect by altering the sulfhydryl enzymes and proteins containing allyl thiol groups in bacteria leading to the disruption of the normal physiological functions and energy metabolism in the bacterial cell [87]. Moreover, byproducts of sulfated glucosinolates, such as indoles, thiocyanates and isothiocyanates resulting from enzymatic hydrolysis, showed strong bactericidal activity against *S. aureus*, *E. coli*, methicillin-resistant *Staphylococcus aureus* (MRSA) and *P. aeruginosa* [88,89,90,91,92,93]. However, the byproducts of sulfated salicinoids yielded due to sulfotransferases activities were not tested previously against the bacterial growth [71].

Furthermore, in this present study, we found salicylate glucoside (**13**) and salicortin (**25**) in the twigs of *S. myrsinifolia*, of which salicortin is typical phenolic glycoside of willow twigs. Salicylate glucoside and salicortin were characterized in several willow species with common occurrence in Finland [14,32], including *S. myrsinifolia* [29,81,94]. Salicylic acid is a plant hormone stored in the plant in the glycosidic form, and it is widely present in higher land plants [95]. Salicortin occurs widely in willow species [14] but was found to occur in particularly high concentrations in *S. myrsinifolia* leaves [96] and bark [81]. In antibacterial screenings performed for our present study, we found that salicortin did not inhibit bacterial growth. This is in line with the findings of other authors. However, salicortin has an immune-modulatory effect and thus anti-inflammatory activity [97].

According to our UPLC/QTOF-MS results, several flavonoids could be detected in the twigs of *S. myrsinifolia*, including naringenin (**28a**), taxifolin (**22b**) and luteolin (**38**). In our screenings, naringenin displayed a weak growth-inhibitory effect (39.1%, Table 1) against *P. aeruginosa*. According to a previous study, naringenin did not inhibit the quorum sensing of *P. aeruginosa* [98]. However, naringenin showed an MIC of 125 µg/mL against MRSA [99] and an inhibition zone diameter (IZD) of 18.1 and 23.0 mm, respectively, against *S. aureus* and *B. subtilis* [100]. In line with our result, no activity was observed against the Gram-negative bacterium *E coli* [100]. Thus, naringenin could play a role in the antibacterial activity of the methanol twig extracts of *S. myrsinifolia* against Gram-positive bacteria. SAR studies have revealed that the hydroxylation of naringenin at positions C5-OH, C7-OH and C4′-OH, as well as prenylation at C8-OH or C6-OH, facilitates the attachment of naringenin to the bacterial cell membrane [101,102,103]. In addition, our study revealed that taxifolin is a good inhibitor of *B. cereus* growth, showing an MIC of 250 µg/mL. Our result is in accordance with Liu et al. [104] who reported that taxifolin is active against *S. aureus*, showing an MIC of 556 µg/mL. Furthermore, in their investigation, Liu et al. [104] found that taxifolin strongly synergizes with the activity of tetracycline, induces morphological changes in the bacterial cell and damages the bacterial cell wall and membrane integrity. Thus, taxifolin could play an important role in the antibacterial activity of the twig extract of *S. myrsinifolia* against *B. cereus* that we have seen in this present study.

Many authors reported that an acetyl group in the special metabolites of plants can lead to the acetylation of bacterial enzymes and thus alterations to protein properties that have a major role in virulence, bacterial metabolism and cell signaling [105,106]. Hence, due to the presence of an additional acetyl group in acetylsalicortin (**35**), it showed a better growth-inhibitory effect against *B. cereus* in our screenings (IC 75%) compared to the core molecule of salicortin (IC 68%) (Table 1). Acetylsalicortin might thus be an important contributor to the antibacterial effects of the *S. myrsinifolia* extract against *B. cereus* that were observed in our study.

Moreover, in this present study, we found that the twig maceration and methanol extracts of *S. starkeana* were rich in antioxidant salicinoids, which could play an important role in its antibacterial activity. However, these results warrant further consideration to quantify the antioxidant capacity of the *Salix starkeana* isolated pure compounds in relation to a combination of compounds in *S. starkeana* extracts and fractions.

## 5. Conclusions

In this paper, we evaluated the antimicrobial activity of polar methanol and water extracts from the dormant twigs of four *Salix* species occurring in Finland, including *S. starkeana*, *S. myrsinifolia*, *S. phylicifolia* and *S. x pendulina*. The growth-inhibitory effects were mild with MIC values from 625 to 2500 µg/mL for the willow extracts. Methanol extracts of the twigs of *S. starkeana* and *S. myrsinifolia* were especially growth inhibitory against the Gram-positive bacteria, with an extract of *S. starkeana* showing the lowest MIC of 625 µg/mL against *B. cereus*. Our report of the good antibacterial effects of *S. starkeana* is the first to the best of our knowledge. According to a time-kill assay, a methanol extract of the twigs of *S. myrsinifolia* (at an 2 × MIC concentration of 5000 µg/mL) was more active than tetracycline (at an 0.5 × MIC of 0.24 µg/mL) against *B. cereus* and showed bactericidal effects throughout the incubation time of 24 h. Our phytochemical results indicate that the dark-leaved willow, *S. myrsinifolia*, twigs contain a high variety of phenolic compounds, including proanthocyanidins and some flavonoids, such as catechin and taxifolin, which inhibited the growth of *B. cereus* with an MIC value of 250 µg/mL. Paunonen et al. [107] suggested that the dark-leaved willow could be a promising source of herbal drugs, which is now supported by our results. Due to its rapid growth, good pest resistance and high content of salicylates and other polyphenols in the leaves and twigs, the dark-leaved willow could be especially suitable for herbal willow cultivation [108].

Altogether, the *Salix* species screened in this study contain a high number of compounds that could have value as scaffolds for new antimicrobials or as antibiotic adjuvants. However, the mechanistic details of how *Salix* extracts and compounds affect bacterial growth and communication (biofilm formation and eradication) are still not clear and should be investigated in detail.

Lastly, from an environmental perspective, the management of *Salix* stands surrounding agricultural fields in Finland should include the more effective use of the naturally occurring *Salix* species. Specifically, the *Salix* management, including the early thinning or advanced thinning (by topping and cutting secondary stems and branches), contributes greatly to reduce ecological competition between the *Salix* species and increases the possible ecological and economic benefits [109,110]. Moreover, for example, the removed twigs and branches from the *Salix* spp. during this thinning process could be used in a sustainable way for various purposes in a circular economy, such as for producing standardized extracts without destroying the *Salix* stands. As for now, in many cases in Finland, the *Salix* stands are cut down completely and used as energy plants without the step in between to extract valuable phytochemicals.

## Data Availability

The data presented in this study are openly available in Helda at https://helda.helsinki.fi.

## Supplementary Materials

The following supporting information can be downloaded at: https://www.mdpi.com/article/10.3390/pharmaceutics16070916/s1, Figure S1. UV spectra of procyanidin B1 and its isomers from a methanol twig extract of *Salix myrsinifolia*; Figure S2. Mass spectra of procyanidin B1 and its isomers from a methanol extract of the twigs of *S. myrsinifolia*. The molecular ion at [M–H]^−^ 577 is visible in all spectra; Figure S3. UV absorption maxima of a procyanidin B1 standard and procyanidin B1 in *Salix myrsinifolia*; Figure S4. Mass spectra of (a) a procyanidin B1 standard and (b) of procyanidin B1 in *Salix myrsinifolia.* The molecular ion at *m*/*z* [M–H]^−^ 577 is typical for the dimeric procyanidin B1; Figure S5. UV absorption maxima of (a) the salicortin standard and (b) salicortin from *S. myrsinifolia*. A UV absorption maximum of 270 nm can be seen; Figure S6. Mass spectrum of (a) salicortin in *S. myrsinifolia* and of (b) the salicortin standard; Figure S7. Mass spectra of salicin in (a) *Salix myrsinifolia* and (b) of the salicin standard. The molecular ion at *m*/*z* [M–H]^−^ 285 is marked with an arrow. Figure S8. UV absorption spectra of (a) a salicin standard and (b) salicin in *Salix myrsinifolia*. Figure S9. UPLC-DAD and total ion current (TIC) chromatogram of methanol twig extract of *Salix myrsinifolia*. Figure S10. UPLC-DAD chromatogram of the standard compounds (a), salicin; (b), procyanidin B1 and (c), salicortin.
