# Supplementary material for: Polyphenols and Phenolic Glucosides in Antibacterial Twig Extracts of Naturally Occurring Salix myrsinifolia (Salisb.), S. phylicifolia (L.) and S. starkeana (Willd.) and the Cultivated Hybrid S. x pendulina (Wender.)
Source: Pharmaceutics. 2024 Jul 9;16(7):916. doi: 10.3390/pharmaceutics16070916 (PMC11280161; doi:10.3390/pharmaceutics16070916)
Supplement: Supplementary file 1 [file pharmaceutics-16-00916-s001.zip › pharmaceutics-3031398-supplementary.pdf]

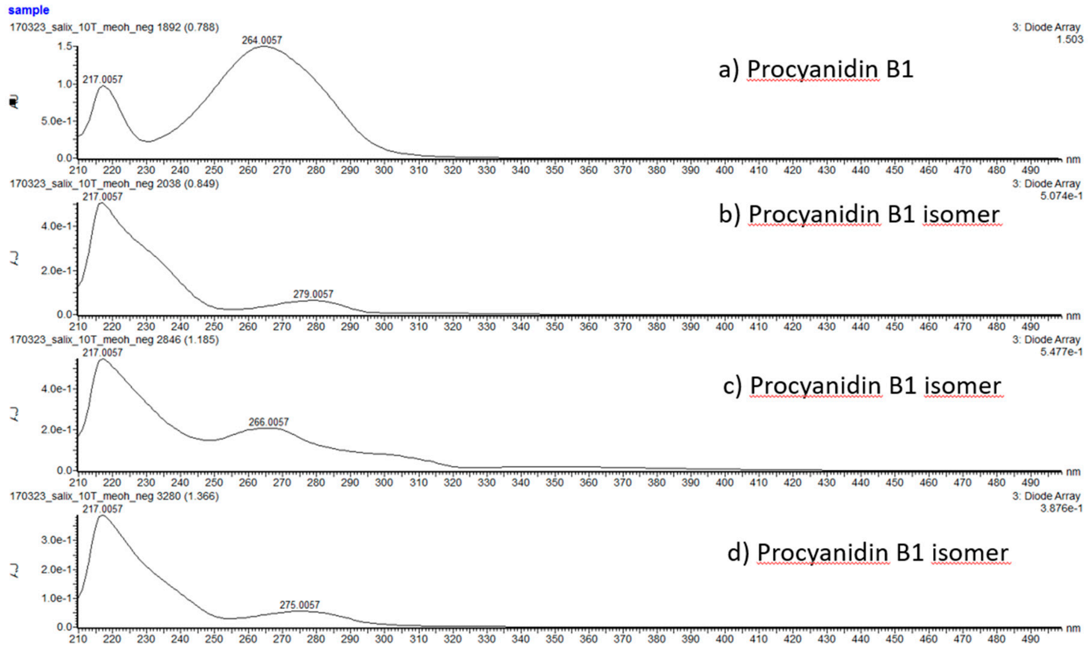

Figure S1. UV spectra of procyanidin B1 and its isomers from a methanol twig extract of *Salix myrsinifolia*.

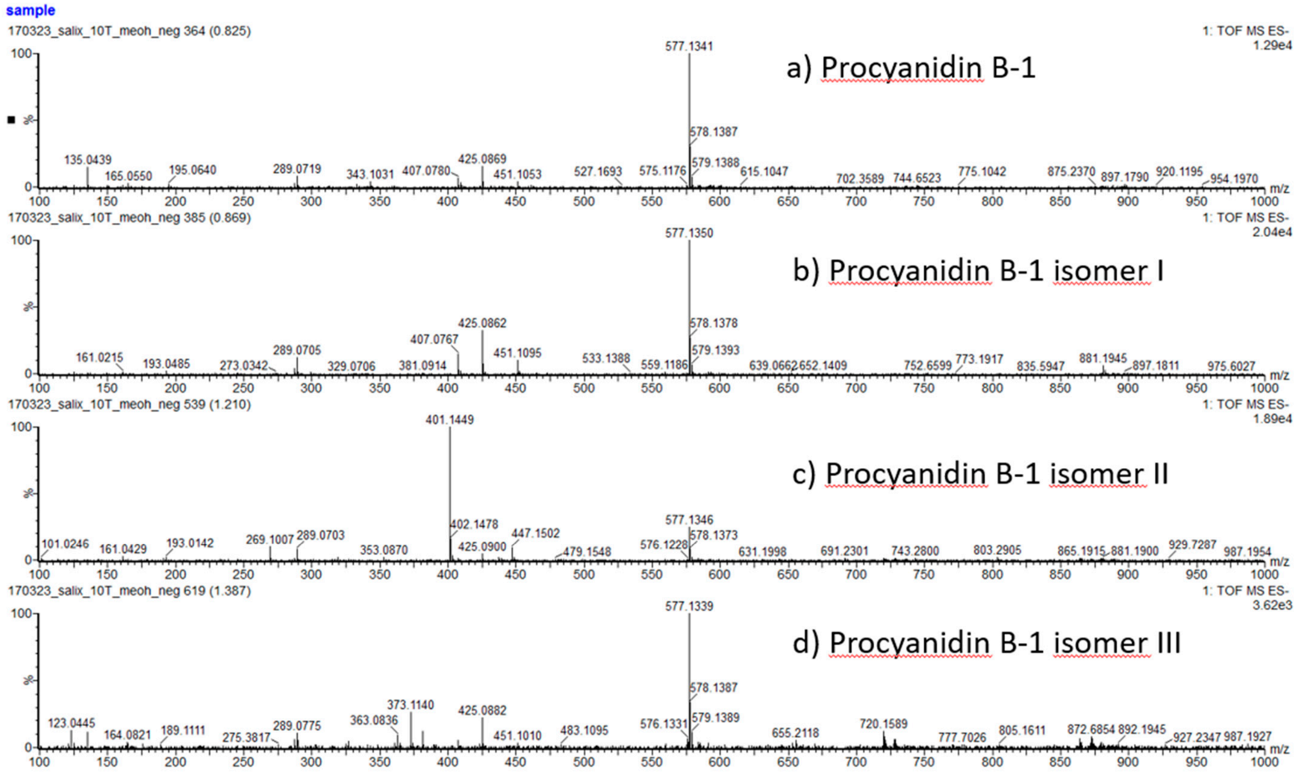

Figure S2. Mass spectra of procyanidin B1 and its isomers from a methanol extract of the twigs of *S. myrsinifolia*. The molecular ion at [M-H]- 577 is visible in all spectra.

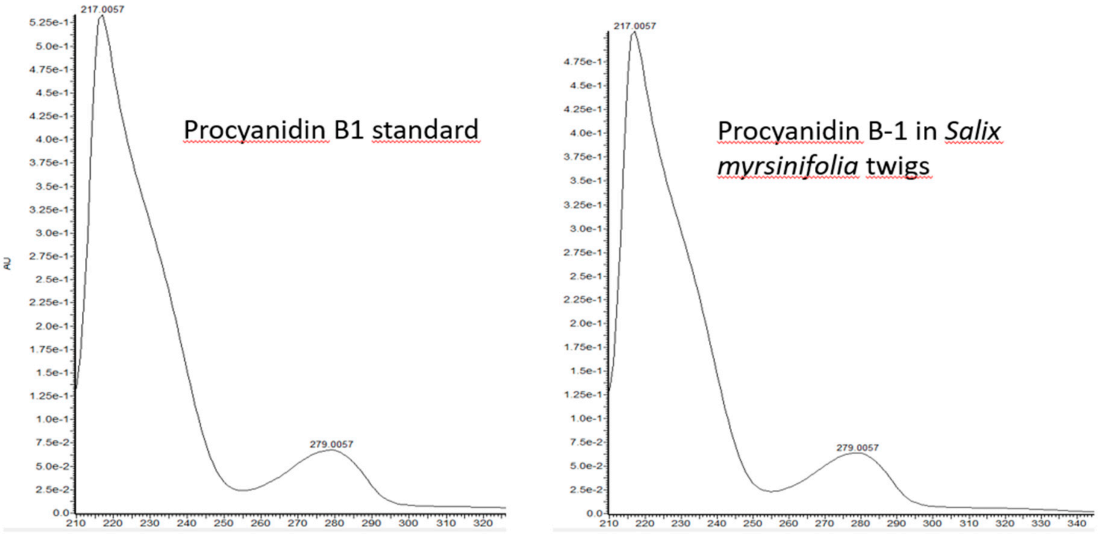

Figure S3. UV absorption maxima of a procyanidin B1 standard and procyanidin B1 in *Salix myrsinifolia*.

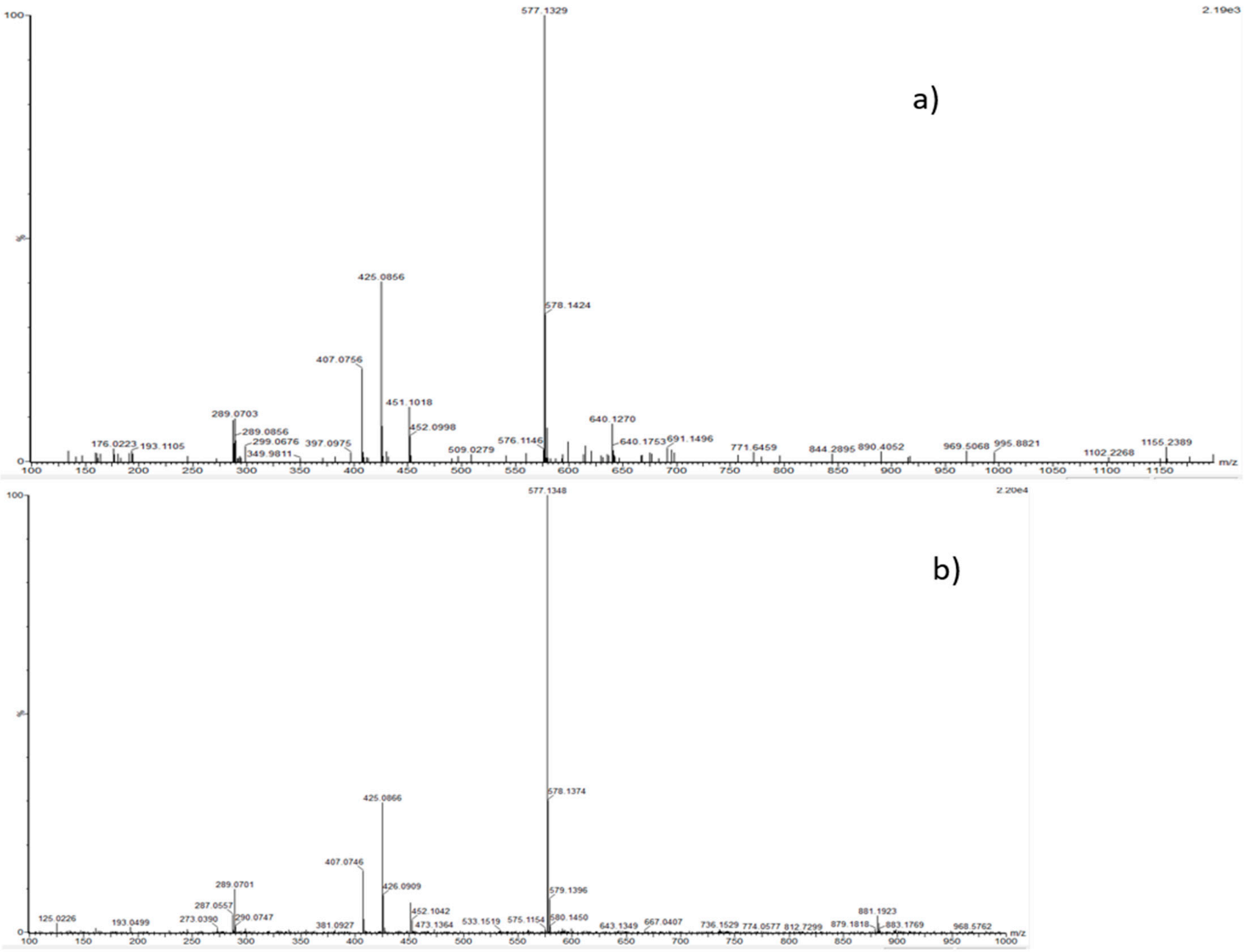

Figure S4. Mass spectra of a) a procyanidin B1 standard and b) of procyanidin B1 in *Salix myrsinifolia.* The molecular ion at m/z [M-H]- 577 is typical for the dimeric procyanidin B1.

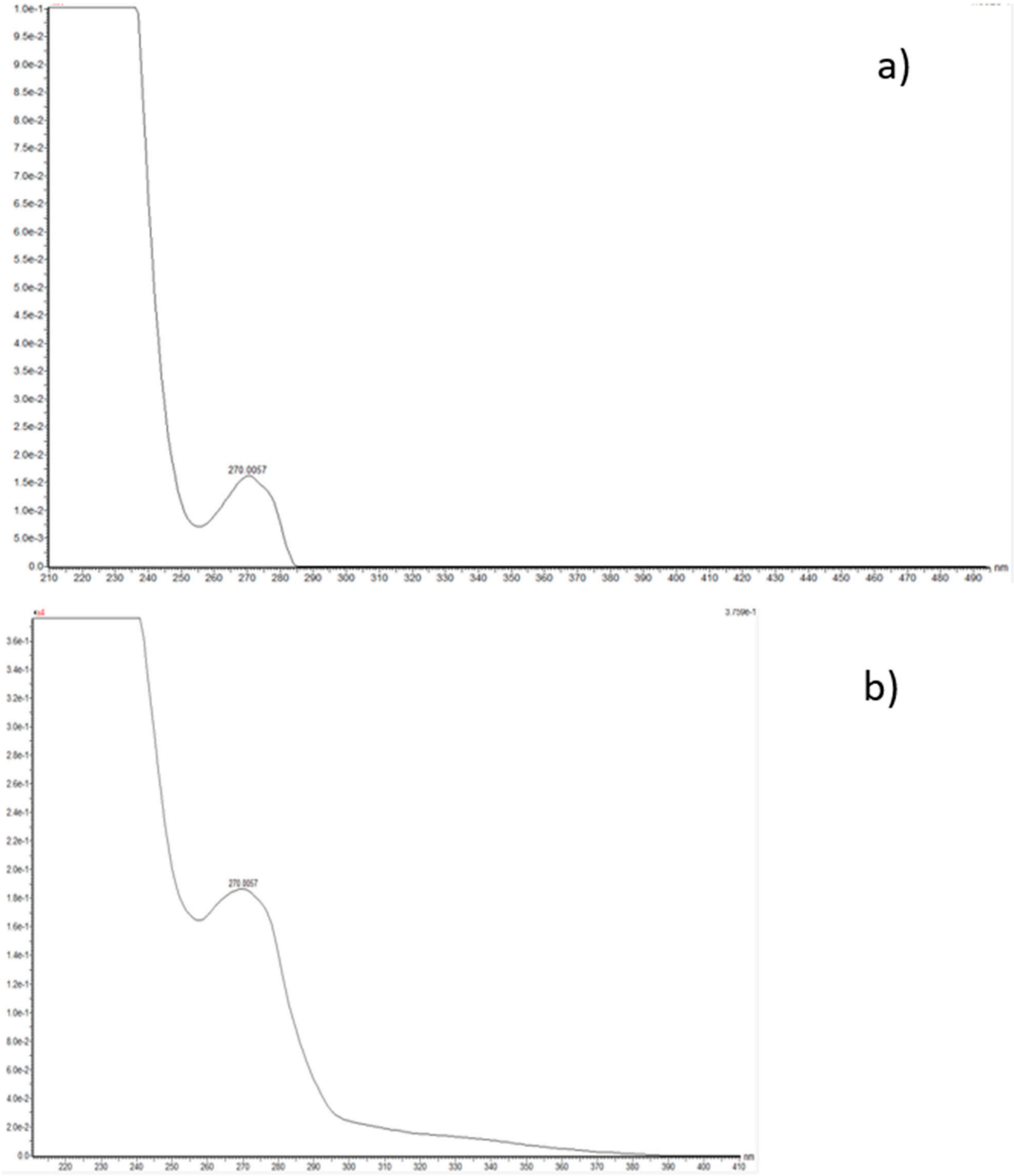

Figure S5. UV absorption maxima of a) the salicortin standard and b) salicortin from *S. myrsinifolia*. A UV absorption maximum of 270 nm can be seen.

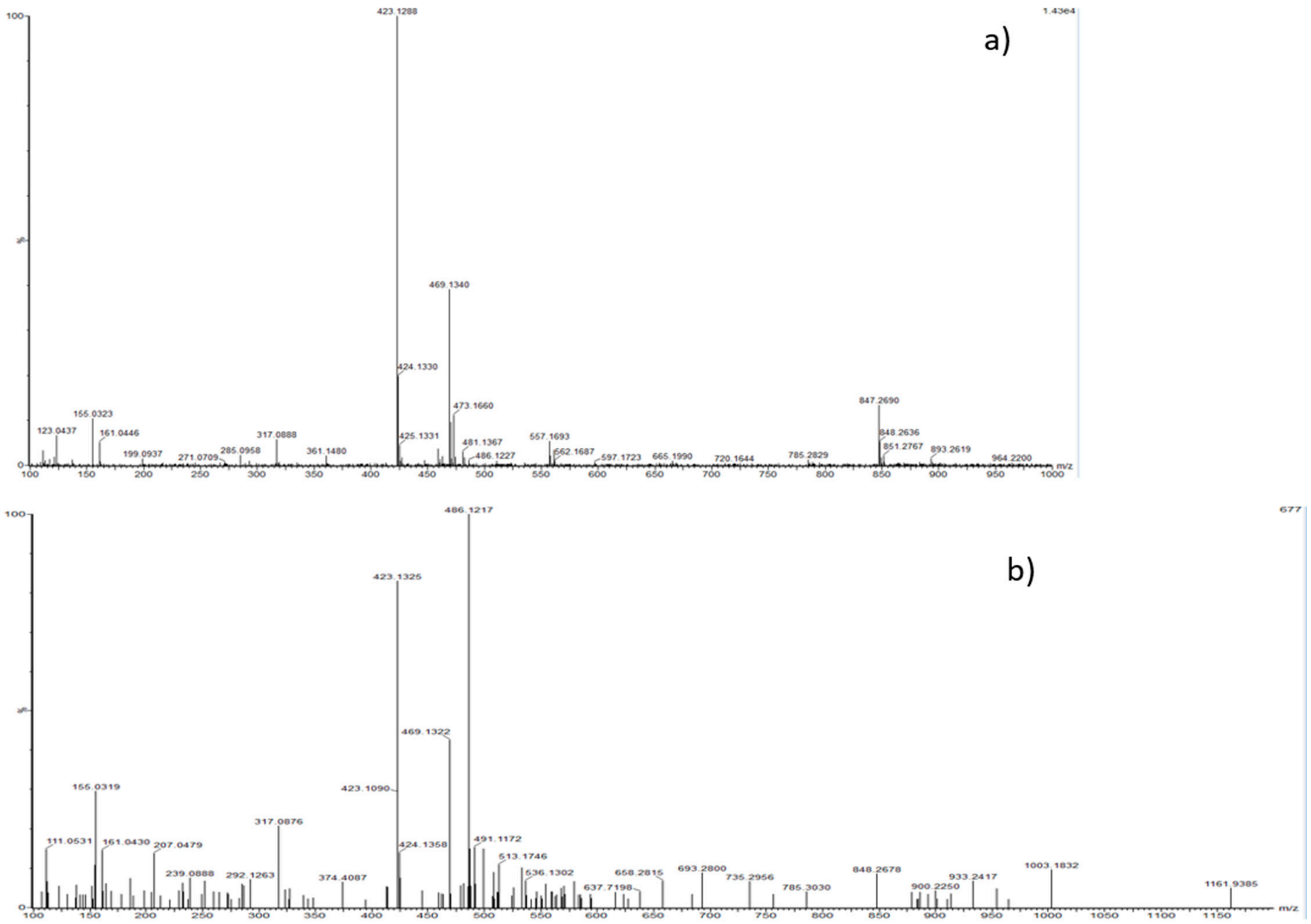

Figure S6. Mass spectrum of a) salicortin in *S. myrsinifolia* and of b) the salicortin standard.

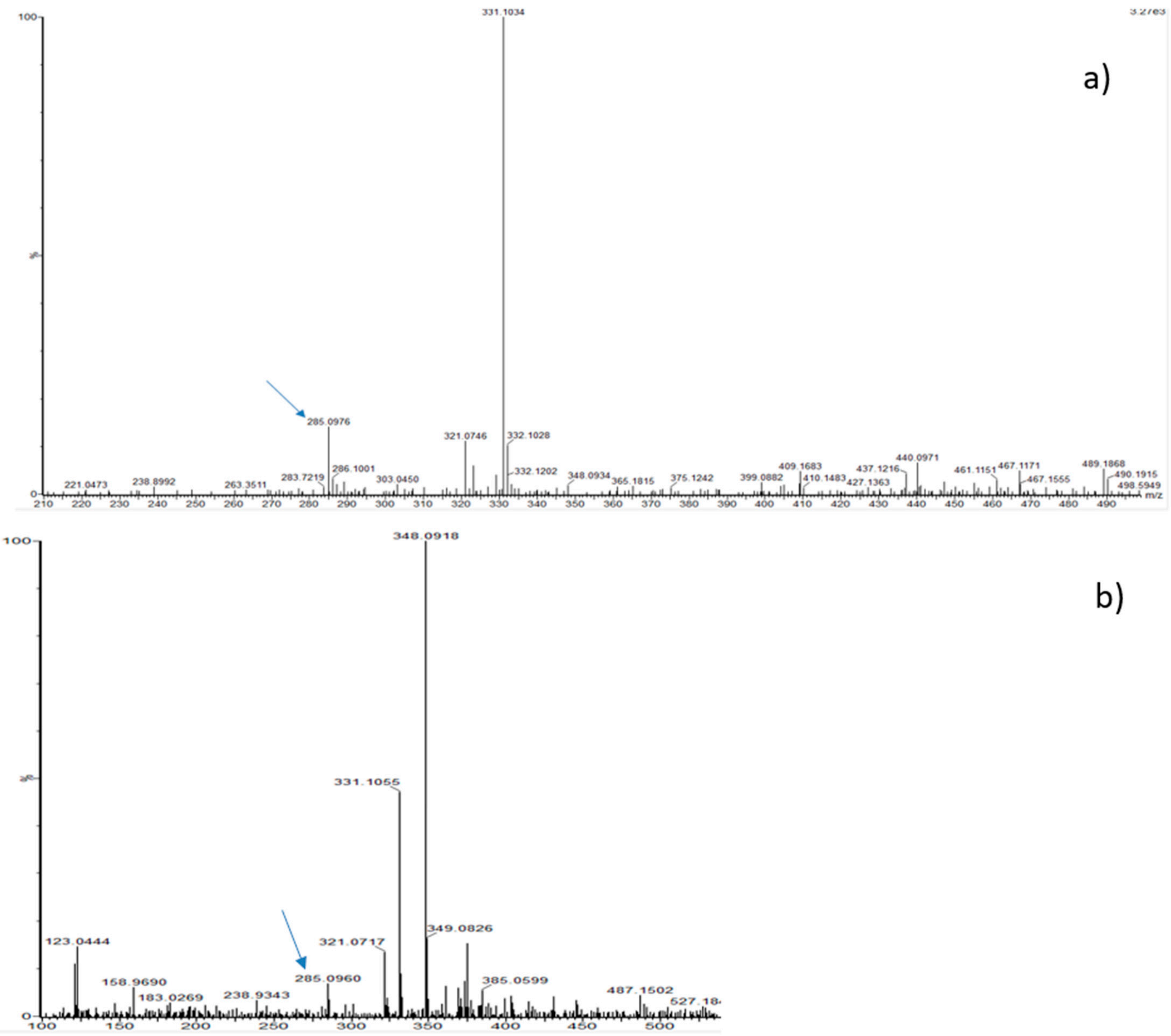

Figure S7. Mass spectra of salicin in a) *Salix myrsinifolia* and b) of the salicin standard. The molecular ion at m/z [M-H]- 285 is marked with an arrow.

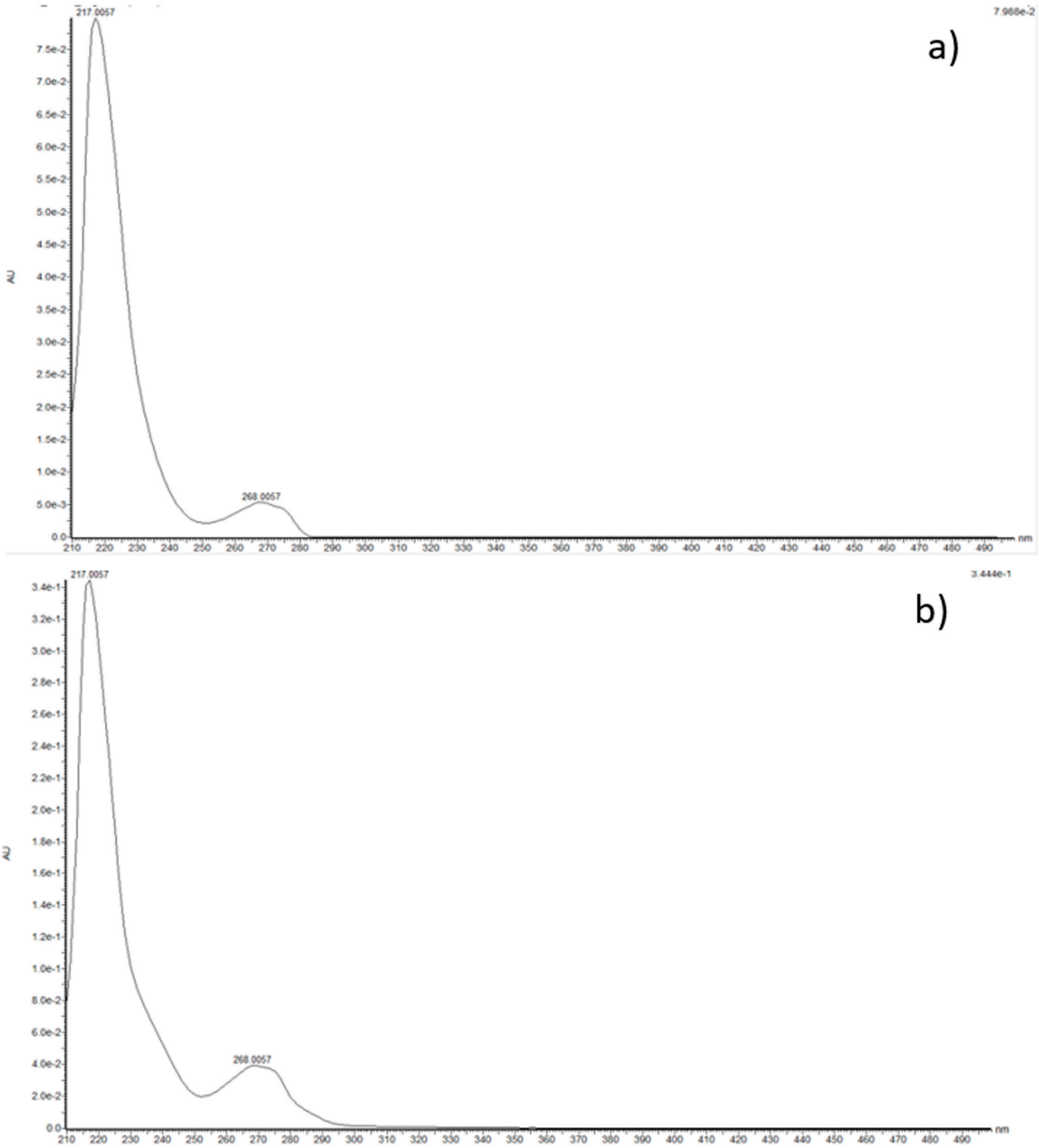

Figure S8. UV absorption spectra of a) a salicin standard and b) salicin in *Salix myrsinifolia*.

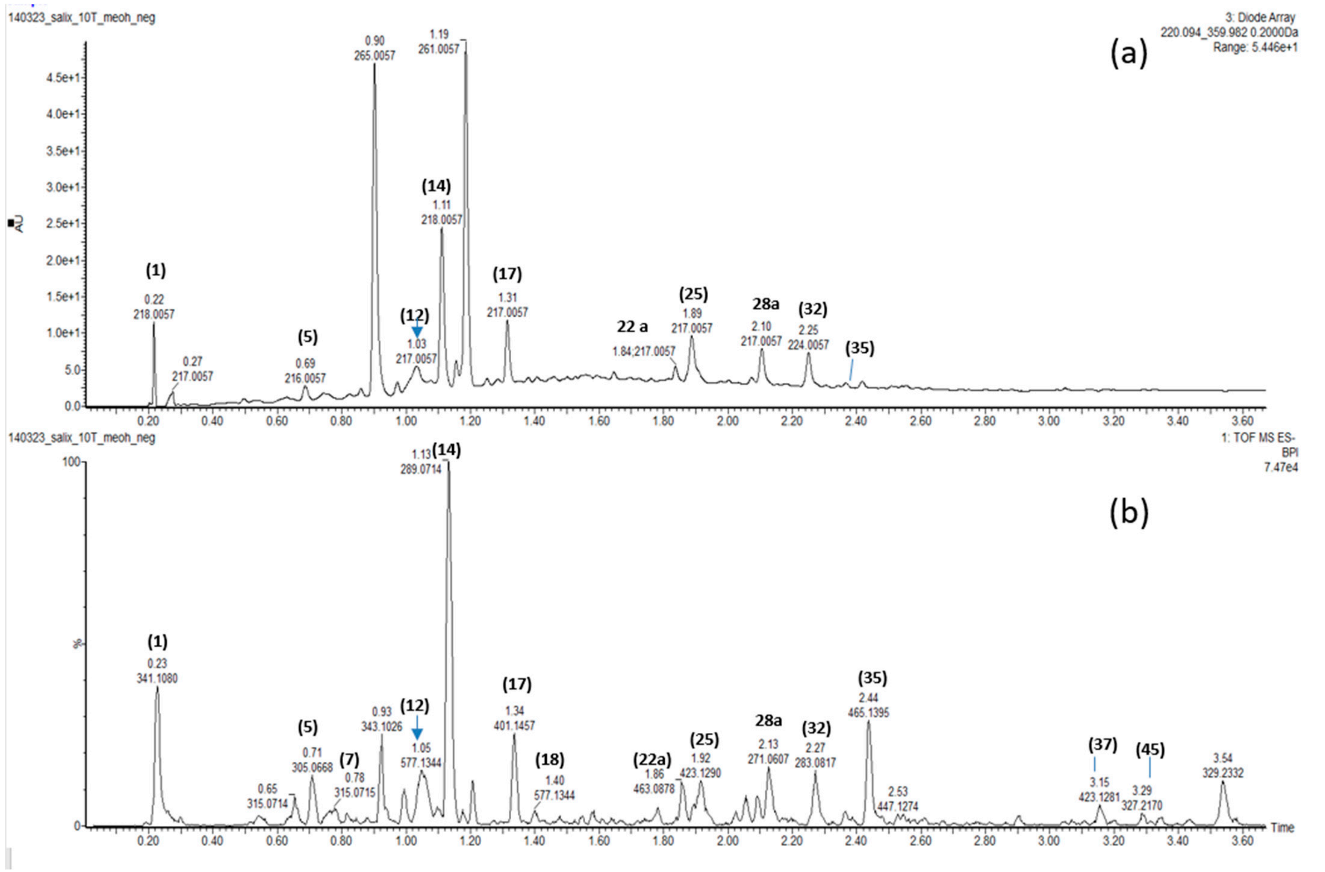

Figure S9. UPLC-DAD and total ion current (TIC) chromatogram of methanol twig extract of *Salix myrsinifolia*. For the UPLC-DAD, diode array detection from 220-360 nm was used. The compounds are numbered as in Salih et al., 2024. (1) Caffeoylhexose, (5) epigallocatechin, (7) protocatechoylglucose, (12) procyanidin B1, (14) catechin, (17) benzyl-ß-primeveroside, (18) procyanidin B1 isomer, (22a) isoquercitrin, (25) salicortin, (28a) naringenin, (32) helicin, (35), acetyl-O-salicortin, (37) salicortin derivative, (45) unknown compound, (47) unknown compound.

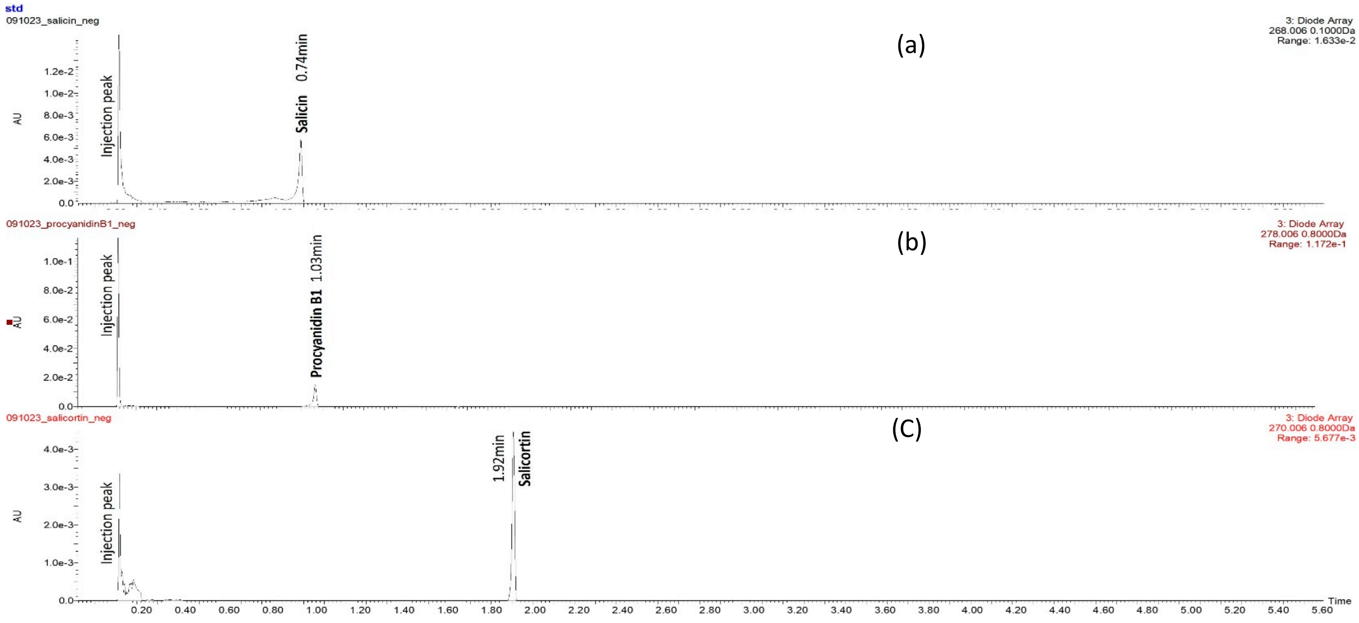

Figure S10. UPLC-DAD chromatogram of the standard compounds (a), salicin; (b), procyanidin B1 and (c), salicortin.
